# Perspective on intermetallics towards efficient electrocatalytic water-splitting

**DOI:** 10.1039/d1sc01901e

**Published:** 2021-06-08

**Authors:** Carsten Walter, Prashanth W. Menezes, Matthias Driess

**Affiliations:** Derpartment of Chemistry: Metalorganics and Inorganic Materials, Technische Universität Berlin Strasse des 17. Juni 135, Sekr. C2 Berlin 10623 Germany prashanth.menezes@mailbox.tu-berlin.de matthias.driess@tu-berlin.de

## Abstract

Intermetallic compounds exhibit attractive electronic, physical, and chemical properties, especially in terms of a high density of active sites and enhanced conductivity, making them an ideal class of materials for electrocatalytic applications. Nevertheless, widespread use of intermetallics for such applications is often limited by the complex energy-intensive processes yielding larger particles with decreased surface areas. In this regard, alternative synthetic strategies are now being explored to realize intermetallics with distinct crystal structures, morphology, and chemical composition to achieve high performance and as robust electrode materials. In this perspective, we focus on the recent advances and progress of intermetallics for the reaction of electrochemical water-splitting. We first introduce fundamental principles and the evaluation parameters of water-splitting. Then, we emphasize the various synthetic methodologies adapted for intermetallics and subsequently, discuss their catalytic activities for water-splitting. In particular, importance has been paid to the chemical stability and the structural transformation of the intermetallics as well as their active structure determination under operating water-splitting conditions. Finally, we describe the challenges and future opportunities to develop novel high-performance and stable intermetallic compounds that can hold the key to more green and sustainable economy and rise beyond the horizon of water-splitting application.

## Abstract 2

This perspective provides an overview of the versatility of intermetallic compounds for electrochemical water splitting along with their synthetic strategies, catalytic efficiencies as well as their active structures under operating conditions.

## Graphical abstract

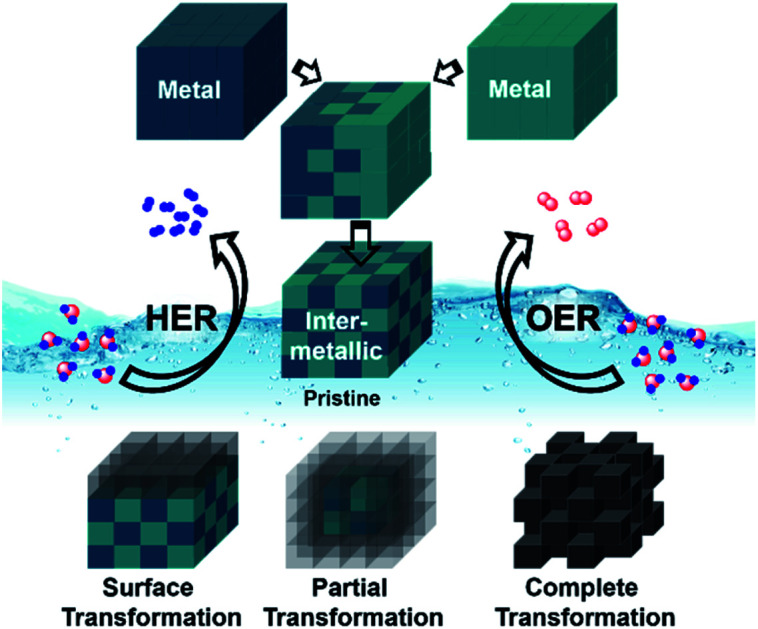

## 1. Introduction

The rising global demand for energy is experiencing major challenges with its exponential growth and nearly entire dependency on fossil fuels.^1^ Depletion of these natural resources and the inconvenient impact on climate with its carbon emissions stipulate alternative strategies in energy supply.^2^ In the last few years, tremendous efforts have been dedicated to fulfil the required energy demand by renewable and green technologies and are expected to continue to grow in the years to come.^3^ In this regard, hydrogen (H_2_) is considered as an abundant and clean fuel that can be used as a chemical energy store with a minimal loss of energy.^4,5^ H_2_ has the highest chemical energy per mass with 143 MJ kg^−1^ (ref. 6) and its energy density is three times higher than that of diesel or gasoline (47 MJ kg^−1^). Once produced, H_2_ is a clean synthetic fuel, energy supplier for households and the economy, and an important raw material for the chemical industry.^6,7^ A practical and attractive approach to produce inexpensive, reliable, and highly pure H_2_ is electrochemical water splitting.^8,9^ Depending on the pH value of the electrolyte, water splitting is realized with a polymer electrolyte membrane (PEM) electrolyzer in acidic conditions, neutral water or with alkaline electrolyzer (AEC), and at high temperatures with neutral water with solid oxide electrolysis (SOEL).^10^ Generally, these technologies are referred to as water electrolyzer cells (WEC) and considered as the most promising technologies for power-to-(P2G) gas conversion.^3,10^ WECs have their own characteristic merits and shortcomings depending on the process conditions, efficacy, materials, and purity of the product. So far, SOEL has shown the highest efficiency of the WEC technologies but demands challenging material properties due to its high-temperature process of 700–900 °C.^10^

Alternatively, PEM technology provides high energy efficiency and fast H_2_ production rate with high purity, however, impedes their catalyst scope mainly to precious metal and metal oxides resulting in the high cost and limiting their widespread industrial application.^10,11^ In comparison to PEM, an AEC is considered the mellow approach and has widely been used as the leading technology in large-scale industrial applications.^10,12^ Mediating H_2_ evolution in alkaline media allows replacing noble metals with earth-abundant materials, that either are not stable in acidic conditions or only show poor activities.^13^ Therefore, AECs have attracted research to explore various 3d transition metals as alternative catalysts for benchmarking noble-metal IrO_*x*_, RuO_*x*_, and Pt systems.^14,15^

Over the years, to bring the WECs to cost parity, numerous classes of cost-effective electrocatalysts such as metal oxides/(oxy)hydroxide,^16^ chalcogenides,^17,18^ pnictides,^19,20^ phosphates,^21^ phosphites,^22^ borophosphates,^23,24^ borides,^25^ selenides,^26^ carbides,^27^ alloys,^28^ and their heterostructures, have been reported either as cathodes or anodes and a majority of them have shown promising results in comparison to the state-of-the-art catalysts. The reason for superior activity is often described as the presence of a higher number of active sites on the surface, better reaction kinetics as well as the modified electronic properties. In this regard, intermetallic compounds possess complex structures with distinct chemical bonding and have already been found applications in magnetism, superconductivity, shape-memory effects, H_2_ storage, and recently even for heterogenous catalysis.^29^ Due to covalent bonds, intermetallics are considered as (electro-)chemical stable with high electric conductivity. The high activity is often related to a high number of active sites resulting from the highly ordered structure with voids.^30^ These enhanced attractive physical, chemical, and electronic properties with suitable element specificity in intermetallic compounds have attracted considerable interest as ideal electrocatalysts for water splitting.

**Scheme 1:**
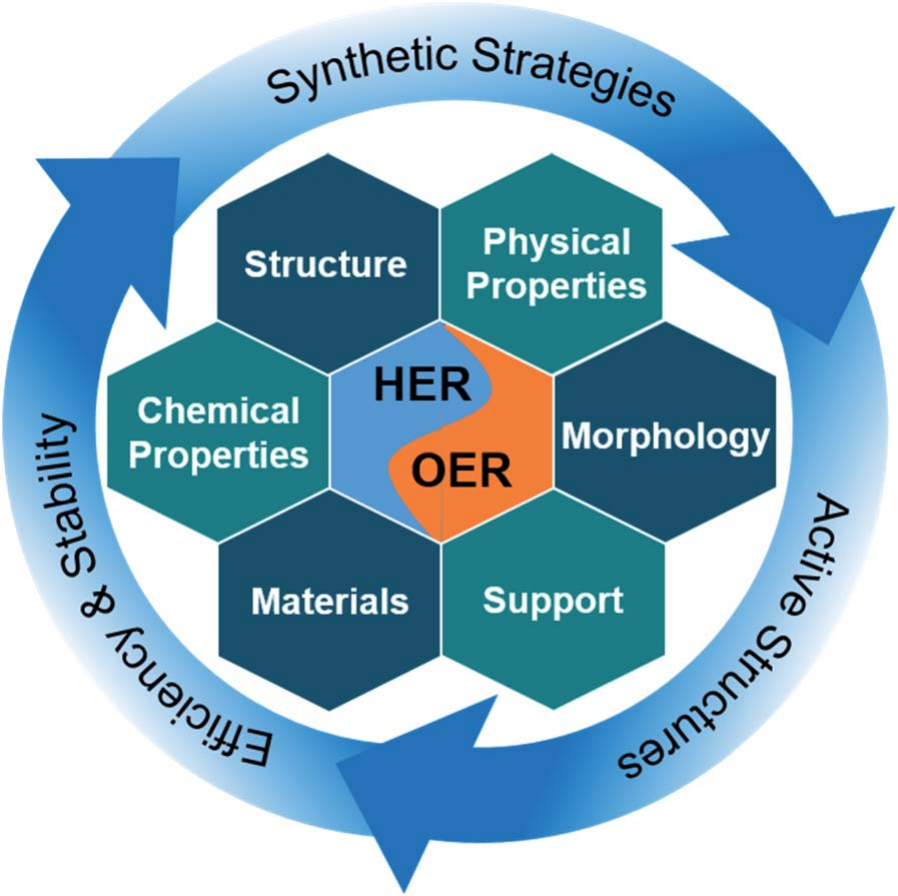
Intermetallics for water splitting describing the synthesis strategies to access intermetallic materials with different morphological, structural, chemical, and physical properties with diverse elemental composition. This includes insights into active structure and the reasons for their high efficiency and stability in electrochemical water spitting.

In this perspective, we focus on the most recent advances in electrocatalytic water-splitting with intermetallic materials (Scheme 1). First of all, we present the fundamental understanding of electrocatalytic water splitting and its evaluation parameters. Next, in order to give the readers a conception of the development methodologies, we discuss the underlying aspects as well as advanced synthetic strategies in detail. Subsequently, we review the electrocatalytic performance of numerous noble-metal and non-noble metal-based intermetallic compounds that have been tested for water splitting in acidic, neutral, and alkaline media. Special emphasis has been given to the understanding of the nature of precatalyst and active catalysts, their modifications, electronic modulations, and structure–activity relation under reaction conditions. In the end, we highlight the future perspectives and challenges of intermetallic compounds for water splitting as well as we provide some proposed topics that are potentially interesting for future research. We consider that this perspective will provide valuable guidelines and deep insights into the field and will also inspire many to research a similar direction.

## 2. Electrochemical water-splitting

For hydrogen production by electrochemical conversion of water to oxygen and hydrogen, the free energy of Δ*G* = 238 kJ mol^−1^ is needed to mediate the reaction (eqn (1)).1
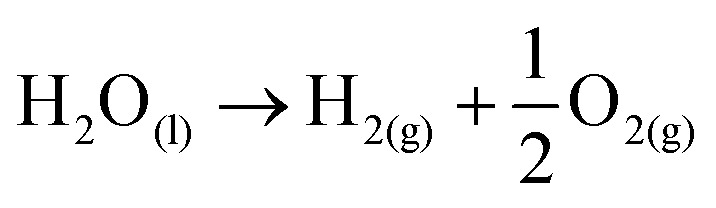


Although it is seemingly a simple straightforward reaction adjunct to plain electrochemistry, the energy loss entangled to mechanistic intricacy demands strenuous efforts to facilitate water splitting.^1,31^

### 2.1 Fundamentals

Water splitting is divided into two half-reactions, water reduction at the cathode for the hydrogen evolution reaction (HER) and water oxidation for oxygen evolution reaction (OER) at the anode.^32,33^

Cathode (HER):

2 2H^+^ + 2e^−^ → H_2_*E*^0^_Red_ = 0.00 V

Anode (OER):3
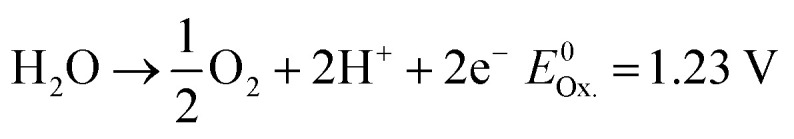


OER in particular requires significantly more energy than HER due to comparably more intermediates and reaction steps.^34^ Accounting for the sluggish uphill reaction, OER is still referred to as the bottleneck of overall water splitting, and that is why, in the last few years, immense efforts have been dedicated to seek alternative materials that can effectively reduce the kinetic limitation of OER and enable optimal reaction conditions.^21^ Both reactions take place at the electrode surface (anode and cathode) together with the electrolyte and require consideration of the inner and outer Helmholtz layers.^35^ The performance of these reactions can be measured separately using electrochemical techniques, which can be used to determine the exact values of the applied potentials.^36^ In this regard, the electrochemical HER and OER can be expressed in terms of two redox pairs. In OER, the reduced form H_2_O and the oxidized form O_2_ form the redox couple (O_2_/H_2_O).^2^ The HER is composed of the reduced form H_2_ and the oxidized form H^+^ to form the redox couple (H^+^/H_2_). Each of both redox systems have different degrees of reducing and oxidizing power as described in the eqn (2) and (3)^2^ and can be described electrochemically by the redox potential *E* of the redox system.^2^ The redox potential can be described mathematically by the Nernst equation (see eqn (13)):^37^4
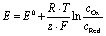


The constant *z* describes the number of electrons occurring in the redox system and *R* is defined as the general gas constant.^36^ The standard potentials are characteristic of each redox system and cannot be determined experimentally. They form a parameter for the strength of the reducing or oxidizing effect of such a system and thus indicate whether electrons are taken up or released.^36,38^ Only the total potential of a galvanic element can be measured and the potential difference between two redox pairs can be determined to a reference.^36^

### 2.2 Reaction mechanisms

**Fig. 1:**
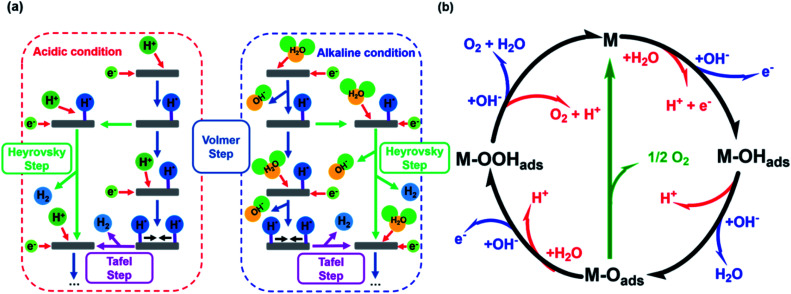
(a) HER mechanism in acidic and alkaline conditions with the Volmer reaction (blue), Heyrovsky reaction (green), and Tafel reaction (purple). Adapted with permission from ref. 39 copyright 2018, Springer Nature (b) OER mechanism for acidic (red) and alkaline (blue) conditions. The black line shows the OER pathway *via* an active site with the M–OOH_ads_ intermediate. The alternative pathway *via* two M–O_ads_ intermediates is shown in green. Adapted with permission from ref. 40, copyright 2017, RSC Publishing.

The HER is kinetically favored and takes place relatively thermodynamically uninhibited with the formation of the single intermediate M–H_ads_ which requires ideally a binding energy of 0 V.^41,42^ Its kinetics are defined by two intermediate steps with three possible pathways, the Volmer, and the Heyrovsky or Tafel step with two-electron transfers (see eqn (5)–(7), Fig. 1a).^32,43,44^

Volmer:

5 M + H_2_O + e^−^ → M–H_ads_ + OH^−^

Heyrovsky:

6 M–H_ads_ + H_2_O + e^−^ → M + H_2_ + OH^−^

Tafel:

7 M–H_ads_ + M–H_ads_ → 2M + H_2_

The second step depends on the surface concentration of active sites. On surfaces with low concentrations of active sites, the Heyrovsky reaction is preferred (eqn (6)). In this case, a second water molecule is adsorbed at the same active site followed by electron transfer and desorption of dihydrogen and a hydroxyl ion.^45^ The Tafel reaction is the dominant mechanism on catalyst surfaces with a high concentration of active sites. Chemical desorption of dihydrogen is mediated by two active sites close to each other. The distance between the two active sites should not be more than the van der Waals radius of two adsorbed hydrogen atoms, the closer they are more likely this reaction path occurs.^45^

The mechanism of the OER is thermodynamically more demanding than for HER with complex kinetics that varies depending on the pH of the electrolyte and the catalyst material (Fig. 1b).^46^ The fourfold is the limiting factor in this reaction, which contains at least three intermediates.^40,47^ There are several pathways discussed in the literature for varying mechanisms under alkaline and acidic conditions.^13,48–51^ For the OER under alkaline conditions, the oxide pathway or the electrochemical oxide pathway with two active sites and single-site catalysis have been established (eqn (8)–(10)).^45,52^

8 M + OH_aq_^−^ → M–OH_ads_ + e^−^

9.1 M–OH_ads_ + OH_aq_^−^ → M–O_ads_ + H_2_O + e^−^

9.2 M–OH_ads_ + M–OH_ads_ → M–O_ads_ + M + H_2_O + e^−^

10 M–O_ads_ + OH_aq_^−^ → M–OOH_ads_ + e^−^

The last step is often described as proceeding concerted, but can also be divided into two intermediate-steps and proceed *via* the M–OO_ads_^−^ intermediate, which determines the overall reaction rate (eqn (11) and (12)).^40,52^

11 M–OOH_ads_ + OH_aq_^−^ → M–OO_ads_^−^ + H_2_O

12 M–OO_ads_^−^ → M + O_2_ + e^−^

The proposed mechanisms for one or two active sites differ only slightly and include the same intermediates as M–OH_ads_ and M–O_ads_.^40,52^ The main difference is the final O–O bond formation and release of dioxygen.^40^ The reaction *via* two catalytically active sites takes place through two M–O_ads_ intermediates with a direct combination of both to form 2M and O_2_ (eqn (13)).

13 M–O_ads_ + M–O_ads_ → 2M + O_2_

Ideally, the binding of the intermediates requires 1.23 eV each, so that free energy of 4.92 eV is required for all four proton-coupled electron transfer (PCET) steps.^2^ All formed intermediates (M–OH_ads_, M–O_ads_, M–OOH_ads_, M–OO_ads_^−^) influence the kinetics of the reaction and have a limiting effect on the reaction rate (higher overpotential) if the adsorption or desorption or formation of the respective intermediate proceeds energetically unfavorably.^52^ The route *via* a single active site with the formation of the M–OO_ads_^−^ intermediate as discussed above is rarely found in the literature but is taken into account for investigations of kinetic barriers during OER.^52^ Despite these differences, there is mutual consensus about the crucial importance of bond interactions in M–O within these intermediates (M–OH_ads_, M–O_ads_, M–OOH_ads_, M–OO_ads_^−^). The M–O bond characteristics of the active species M are essential for the overall electrocatalytic activity.^40^

### 2.3 Activity evaluation criteria

The activity parameters are the most important criteria to evaluate the activity, efficiency, and stability of a catalyst. The overpotential of an electrochemical process is the additional potential required to catalyze the intended reaction at the thermodynamically determined reversible potential (*E*^0^) under ideal conditions.^53^ Typically the overpotential *η* at a current density of 10 mA cm^−2^ (*η*_10_) derived from CVs or LSVs is used as a benchmark to compare the activities of electrocatalysts and corresponds to the ∼10% solar-to-chemicals efficiency.^54,55^ On the other hand, because of the tremendous growth in the field, researchers have now started to evaluate and compare *η*_100_ and *η*_500_ in addition to *η*_10_ which is highly recommended if a catalyst reaches high current densities. In this way, the evaluation of a catalyst activity would be closer to industrial application standards.

The description of the catalyst activity *via* the Tafel slope provides a lot of information about the properties of the catalyst material and is recommended to measure under steady-state conditions.^56^ The Tafel slope can provide insights into the dynamics of the catalytic processes taking place on the surface and provide information about kinetics and inhibitions during the water oxidation catalysis.^52^ It indicates how much the potential has to increase in order to increase the resulting (measured) current *j* by an order of magnitude.^52^ It is an indication of how efficiently and dynamically an electrode or the catalyst applied to it, reacts to an applied potential and generates a catalytic current. This also takes into account any changes in the mechanism with different *η* and can be used to determine which of the PCET reaction in the mechanism is the rate-determining step.^2,45,57^

During the HER, the Volmer step is the RDS when the reaction is defined by the adsorption and discharge of H^+^ on the catalyst surface, which is indicated by a Tafel slope of ∼120 mV dec^−1^ (eqn (5)).^45^ In the case of a low H_ads_ concentration on the surface, the Heyrovsky step is dominant (eqn (6)). A second H^+^ will be adsorbed at the same active site followed by discharge and desorption of H_2_ and is indicated by a Tafel slope of ∼40 mV dec^−1^. If the H_ads_ concentration on the surface is high, a direct combination of the intermediates is possible to generate H_2_ (eqn (7)) and resulting in a Tafel slope of ∼30 mV dec^−1^.^52^ For the OER process, a Tafel slope of 120 mV dec^−1^ indicates that the overall reaction kinetics is dominated by the coordination of the hydroxide step (eqn (8)).^57^ A Tafel slope of 40 mV dec^−1^, indicates the RDS of the electrochemical formation of M–O_ads_ (eqn (9.1)).^57,58^ Alternatively the M–O_ads_ formation can proceed *via* two metal centers indicated by a Tafel slope of 30 mV dec^−1^ (eqn (9.2)). If the reaction is determined by the formation and desorption of O_2_, a Tafel slope of 15 mV dec^−1^ is expected (eqn (13)).^57^

On the other hand, theoretical modelling has suggested that a Tafel slope of 120 mV dec^−1^ is not necessarily accompanied by the Volmer step as RDS. Kinetic studies based on microkinetic analysis gave the same Tafel slope for different elementary steps based on intermediate coverage on the surface and mass transport effects. Especially considering newly proposed radical coupling mechanisms rather than a single-site mechanism for OER.^52^ Further, Liu and co-workers suggested based on calculations that the thermodynamic-kinetic model is also highly dependent on factors such as binding environments, temperature, and electrolyte pH that should also be accounted into the design strategy for catalysts.^59^

Electrochemical impedance spectroscopy (EIS) is also a powerful tool to investigate mechanisms in electrochemical reactions, charge transfer processes in materials, and surface properties of electrodes.^60^ It provides very precise results on the electrical conductivity of a material and is a frequently based technique.^38^ From the obtained impedance spectra, conclusions can be drawn about the ability of materials to store electrical energy and transfer electrical charge.^61^ The electrodes are measured with a two or three-electrode set-up where the potentiostat transmits an alternating potential with varying frequency (*ω*) to the sample. By generating a signal proportional to the generated current. An analyzer then determines the impedance *Z* of the system from the alternating current flowing through the sample and the alternating voltage generated by a generator.^61^ Similarly, the determination of the electrochemically active surface (ECSA) is an *in situ* method, which determines the number of active centers responsible for the respective reaction on the surface (*A*_ECSA_) and thus their size.^62–64^ Other *ex situ* methods such as BET^65^ measurement (BET = Brunauer Emmett Teller) to determine the surface area by adsorbing gas molecules such as nitrogen (N_2_) can be problematic particularly in the case of porous structures since the accessibility to the interior of the structure depends on the size of the adsorbed molecule.^62^ Surfaces determined with N_2_ can be smaller than with krypton or water and provide different results than surfaces determined with ECSA.^62,66^ Both analytical methods to evaluate the surface of the catalyst have their own merits and disadvantages and should be considered carefully. While the BET does not necessarily correspond to the ECSA and could result in an unfair comparison between catalysts with different surface densities of active sites, the ECSA analysis suffers from inaccuracies due to the yet non-trivial determination of the roughness factor.^13^ Therefore, it is highly recommended to represent activity plots normalized to both BET and ECSA. In addition to the activity indicators, the long-term stability of the system is also a crucial parameter considering their use for commercial applications. This is typically measured *via* cyclic voltammetry (CV), galvanostatic chronoamperometry (CA) or potentiostatic chronopotentiometry (CP) electrolysis measurement.

Another valuable insight is the faradaic efficiency (FE), which is defined as the efficiency of electron transfer provided by the external circuit to promote the electrochemical HER or OER reaction. At a constant current density applied for a certain period of time of the experiment, a gas sample is taken with a gas-tight syringe and analyzed with a gas chromatograph (GC) calibrated for H_2_ or O_2_. The faradaic efficiency is then calculated from the volume of the generated gas during electrocatalysis, in comparison to the current over time and gives directly correlates the number of electrons needed to generate a mole of gas.^67–69^ The turnover frequency (TOF) is another important descriptor to evaluate the catalytic activity which provides the generated H_2_ or O_2_ molecules per second at a single active site. However, the precise determination of TOF remains challenging due to the complexity involved in identifying the total number of such active sites and therefore, only a rough estimation is possible.^15^

By calculations of density functional theory (DFT), potential materials suitable for HER and OER can be identified and sorted according to their catalytic activity based on the adsorption (ΔEH) and binding energy (Δ*G*) of the above-discussed intermediates.^70,71^ According to the derived calculations from the DFT, a close relationship between the overpotential and Δ*G* of the surface adsorbed intermediates can be expressed. With a few exceptions, DFT provides good predictions about the OER and HER activity of the materials.^3^ For instance, if Δ*G*_H_ads__ ≈ 0 then the material should possess the optimal binding strength for the intermediate and is considered as a good catalyst for HER. But if is Δ*G* < 0, H_ads_ is bound too strong and desorption of H_2_ is the RDS shifting kinetics *via* the Heyrovsky or Tafel mechanism.^53^ A Δ*G* > 0 indicates a too weakly bounded H_ads_ and therefore, the kinetics is limited by the adsorption of the intermediate, and the reaction proceeds *via* the Volmer step.^53^ DFT calculations have also been applied to study the relationships of Δ*G* in each individual reaction pathway and the activity for both alkaline and acidic media.^72^

## 3. Introduction to intermetallics

**Fig. 2:**
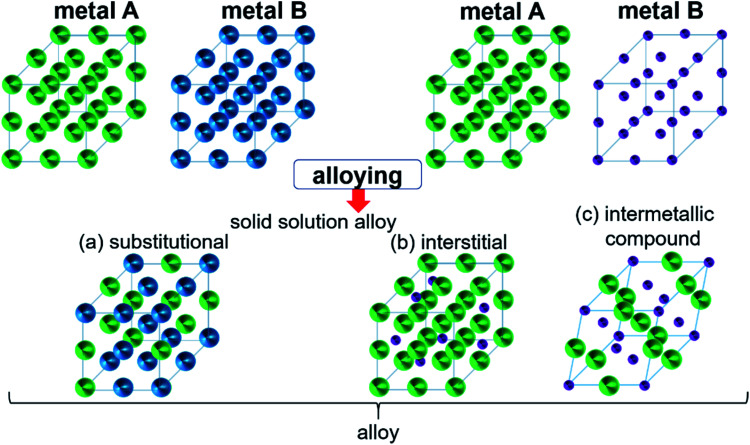
Formation of structures of bimetallic compounds in a solid phase when alloyed: (a) substitutional and (b) interstitial solid-solution alloys and (c) intermetallic compound. Adapted with permission from ref. 29, copyright 2016, American Chemical Society.

Intermetallic compounds belong to the class of alloys but are distinct by the difference between the subclasses on how the atoms are ordered in the crystalline phase.^73^ The bonding in intermetallics involves the combination of partly ionic or covalent interactions instead of weak metallic bonds making them an ordered lattice. Depending on their structure, alloys are initially classified into two different categories: as a solid solution or as an intermetallic compound (Fig. 2).^29^ Solid solutions alloys with metal species of similar atomic radius, electronic character, and crystal structure identical to that of the parent metal form a substitutional solid solution with a statistical distribution of the atoms on the lattice sites (Fig. 2a).^29^ If the atomic radius of an element is sufficiently small to fit into the lattice spaces of the parent metal, an interstitial solid solution forms (Fig. 2b). In these cases, the composition in the degree of mixing can vary almost at will.^29,74^ Based on a linear interpolation between the properties of the parent materials, the same physical properties can be expected in the resulting alloy.^74^ The situation is different when two metals A and B to form an intermetallic compound (Fig. 2c). Intermetallic phases are specified by a clearly defined stoichiometric composition of the elements such as AB, AB_2_, A_3_B, or even complex mixtures such as A_6_B_23_.^74^ Ideally, the metals A and B are not randomly distributed but are arranged at specific positions in the unit cell and differ for A and B.^74^ This often results in physical properties that are distinct from the physical properties of the composite elements with a well-ordered crystal structure disparate from those of the parent elements.^74,75^ Hence, a semiconductor can be derived from two metallic conductors in an intermetallic phase, or a magnetic intermetallic phase can be formed from non-magnetic composing materials.^74,76^

Intermetallic systems have the advantage that a heterogeneous catalytic process such as the adsorption of reactants on the catalyst surface or the activation of reactants mediating the reaction to the desired target product can be assigned to the suitable incorporated species which can cooperate *via* so-called interfaces in the composite material. When it comes to composite materials, intermetallics have a particularly large number of interfaces between the different types of atoms in their structure. A particularly pronounced synergism between activation of the reactants on the surface by one metal and realization of the reaction by fine-tuning of the redox potential with the help of the other can be achieved.^77^ The synergetic effect of the two metallic components improves the properties compared to the individual homometallic components.

Recently, it has been shown that most of the compounds containing anions/nonmetals are unstable under water splitting conditions (especially in OER) and depending on the testing period, they transform either partially or completely to their corresponding mostly layered oxide/oxyhydroxide structures. Such surface/bulk transformed materials from non-oxidic materials have proven to be more active compared to the bare oxide materials due to either high ECSA leading to higher accessible active sites or better electronic conductivity. As the intermetallic compounds are element specific with complex crystal structures and can be formed by both active and conducting elements, they are naturally considered good candidates for electrocatalytic water splitting.

## 4. Chemical synthetic strategies of intermetallics

Most of the so far known 6000 binary intermetallics are mainly derived from solid-state techniques since they have been primarily investigated for their physical and structural properties.^74,76,78^ Intermetallic compounds often show increased stability, selectivity, and activity for a variety of catalytic processes due to their complex structure and bonding characteristics,^73,76,79^ and therefore, over the years, many new advanced synthetic approaches to control their particle size, shape, morphology, and surface area have been developed to gain wider adoption.^30,76^ In the following, several important strategies for the synthesis of intermetallics, starting from single crystals to nanostructures, towards catalytic HER and OER have been discussed in detail.

### 4.1 High-temperature solid-state methods

A historical approach to the synthesis of intermetallics is the thermal annealing method in a protective atmosphere or evacuated conditions at relatively high temperatures to produce thermodynamically stable products in large single crystals from μm up to mm scale.^80^ Though little control remains of the reaction pathway during synthesis (temperature, pressure), and often exploratory efforts are required to attain the desired phase-pure and functional compounds.^80,81^ Giving access to a broad variety of metallic alloy and intermetallic phases, this method is often used to study materials for their physical, chemical, and mechanical properties.^82–86^ The benign technique to develop and predesign intermetallic precursors for the catalytic application has given rise to a large number of water-splitting electrocatalysts.^87–90^

For instance, Lasia and co-workers synthesized Ni–Mo^91^ and Ni–Mo–Al^92^ based intermetallic phases for HER of various compositions by mixing the elemental powders in stoichiometric amounts and melted under an inert atmosphere. While mixing Ni with Mo gave phase pure Ni_4_Mo and Ni_3_Mo, the Ni–Mo–Al phase was a mixture of Ni_2_Al_3_ or Ni_2_Al_3_/NiAl_3_ with NiAl_5_Mo_2_.^91,92^ Similarly, a phase pure Ni_3_Al was prepared in vacuum at temperatures between 600–1250 °C by the group of Liu using commercially available Ni carbonyl and gas atomized Al powder as precursors. The resulting material of a highly porous morphology showed excellent performances in HER catalysis.^93,94^ Meanwhile, Ni_3_Al prepared by Han *et al.* in vacuum at a temperature of 1000 °C using the induction melting technique showed good activity during electrocatalytic OER.^95^

Along this direction, mixing elemental Al and Co metal powders followed by heating in the Ar atmosphere resulted in the formation of an intermetallic nanoporous Al_9_Co_2_ framework that after additional sulfurization acted as a highly efficient precatalyst for HER.^96^

**Fig. 3:**
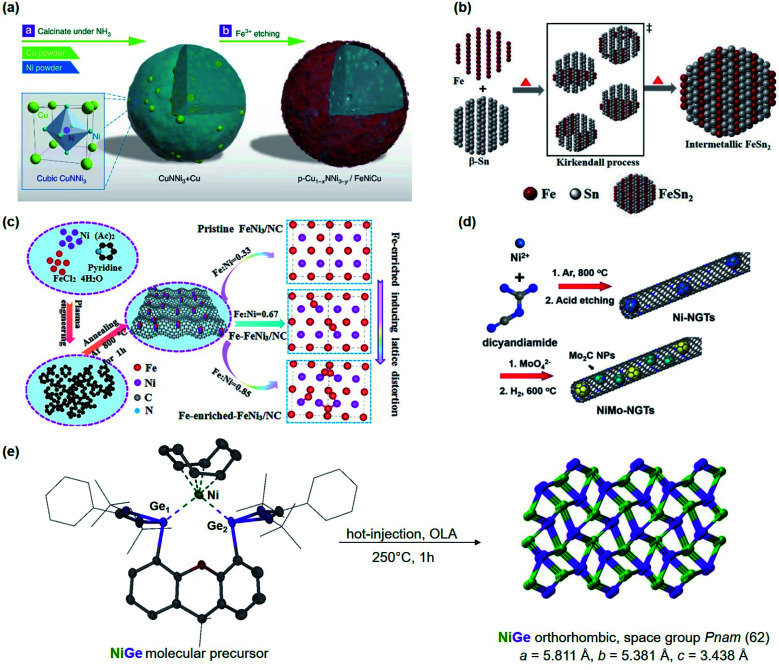
(a) Schematic representation of the synthesis of p-Cu_1−*x*_NNi_3−*y*_/FeNiCu by solid–gas reactions of CuNNi_3_ + Cu and NH_3_ followed by Fe^3+^ etching. Reprinted with permission from ref. 99, copyright 2018, Nature Publishing Group (b) illustration of the formation of intermetallic FeSn_2_ and close packing of the individual atomic layers of Fe and Sn (tetragonal, *I*4/*mcm*) *via* the Kirkendall process. Adapted with permission from ref. 102, copyright 2020, Wiley-VCH (c) synthesis and the fabrication procedure of FeNi_3_/NC, Fe–FeNi_3_/NC and Fe-enriched-FeNi_3_/NC. Reprinted with permission from ref. 103, copyright 2020, Elsevier (d) illustrated synthesis protocol of NiMo-NGTs. Reprinted with permission from ref. 104, copyright 2016, American Chemical Society (e) synthesis of NiGe from the molecular bis(germylene)-Ni complex. Adapted with permission from ref. 105, copyright 2020, Wiley-VCH.

Motivated by the previous results, we recently synthesized polycrystalline MnGa_4_ by annealing a stoichiometric mixture of Mn and Ga in an evacuated quartz ampule to 900 °C for four days to ensure homogeneity of the mixture, then cooled down and annealed at 380 °C for further ten days.^97^ Identical conditions were also chosen to prepare Fe_6_Ge_5_ from Fe powder and Ge chips where the stoichiometric amount of metals were sealed at 1000 °C for two days before annealing again at 650 °C for seven days.^98^ Both intermetallic phases were examined for catalytic OER in alkaline conditions that showed significant activity and stability.^97,98^ In addition, through solid–gas reaction from a mixture of copper and nickel powders, a highly conductive antiperovskite-based hybrid Cu_1−*x*_NNi_3−*y*_ was synthesized and after Fe^3+^ treatment, the formation of a p-Cu_1−*x*_NNi_3−*y*_/FeNiCu with a core–shell structure was observed. Such rational design of the catalyst displayed high conductivity and porosity resulting in a remarkable activity towards OER (Fig. 3a).^99^

The solid-state techniques have been further extended for the preparation of intermetallic phases containing five or more metal elements with high entropic order. Jin *et al.* were successfully synthesized Al_97_Ni_1_Co_1_Ir_1_ and Al_96_Ni_2_Ir_2_ by melting using an induction-melting furnace under Ar protection.^100^ Subsequently, the prepared phases were melted again in a quartz tube and injected onto a spinning Cu roller to prepare the ribbons that were then chemically etched in a 0.5 M NaOH solution to prepare nanoporous high-entropy alloys (np-HEAs) for the HER catalysis.^100^ Very similarly, Ding *et al.* designed a quinary FeCoNiCrNb_0.5_ from their corresponding elemental powders. The eutectic high entropy alloy (EHEA) with a porous nanostructure and high corrosion resistance was successfully applied as a catalyst for OER.^101^

Apart from using traditional induction furnaces and overcome their limitations, arc-melting became a viable method to rapidly (re)melt and refine structures as well as remove high and low-density inclusions during melting.^106^ It is a quite simple and straightforward technique and has widely been applied to attain various noble, *i.e.* PtDy,^107^ Al_2_Pt^108^ and non-noble metal-based, *i.e.* Ti_2_Ni,^87^ TiCo_2_,^109^ Ni_2_Ta^110^ intermetallic compounds for HER and OER. Along this line, lamellar nanostructured Ni–Co–Al was prepared by arc melting the homogenized raw materials at 1200 °C under Ar atmosphere that showed convincing results for alkaline HER.^111^ In a similar approach, the noble metal-based Hf_2_B_2_Ir_5_ phase was obtained *via* a two-step process in which the metals were first arc melted in Ar at about 1200 °C for several weeks and then applied through spark plasma sintering (SPS) to retrieve the electrode material for OER catalysis.^112^ Moreover, in a unique strategy, Jian and co-workers synthesized nanoporous hybrid Cu_12−*x*−*y*_Co_*x*_Mo_*y*_Al_88_ (*x* = 0 or 3, *y* = 0 or 1) electrodes by arc melting pure Cu, Al, Co, and Mo metals in an Ar atmosphere and successively chemically-etched them in a N_2_-purged 6 M KOH electrolyte at 70 °C for 3 h. The synthesized electrodes were successfully applied for overall water splitting, while the OER electrodes were additionally electrochemically oxidized in a dealloying step at 1.57 V *vs.* RHE for 20 min under alkaline conditions.^113^

### 4.2 Chemical reduction

A relatively mild approach at ambient pressure and low temperature is the chemical reduction of metallic precursors in adequate solvents. Varying the reducing agents from the low reducing power of ethanol and linoleic acid to strong with NaBH_4_ and N_2_H_4_ gives control over unwanted reactions where noble metals have dominated the reduction process leading to core–shell structures. With the additional use of surfactants and the specific choice of solvents, various noble and non-noble nanoparticles (NPs), *e.g.* FeIr_3_, NiPd, CoPd_2_, CuRh, NiPt, *etc.*, can be prepared, with varying size and shape from hexagonal or star-shaped to spherical and even small network-like structures.^115–117^ Compared to solid-state, this approach allows overcoming the kinetics energy barrier in the disorder-to-order transition at lower temperatures (<350 °C) allowing structure transition into a well-defined nanoparticular system with proposed composition.^118^ To improve the catalytic activity of catalysts for water splitting, the active sites must have an optimal balance between adsorption and dissociation of intermediates, high electronic conductivity, cost, and stability. Therefore, the chemical reduction is an efficient technique to modulate the electronic structure, reduce or inhibit noble metal content, modify morphology and surface on the atomic level.^118^

**Fig. 4:**
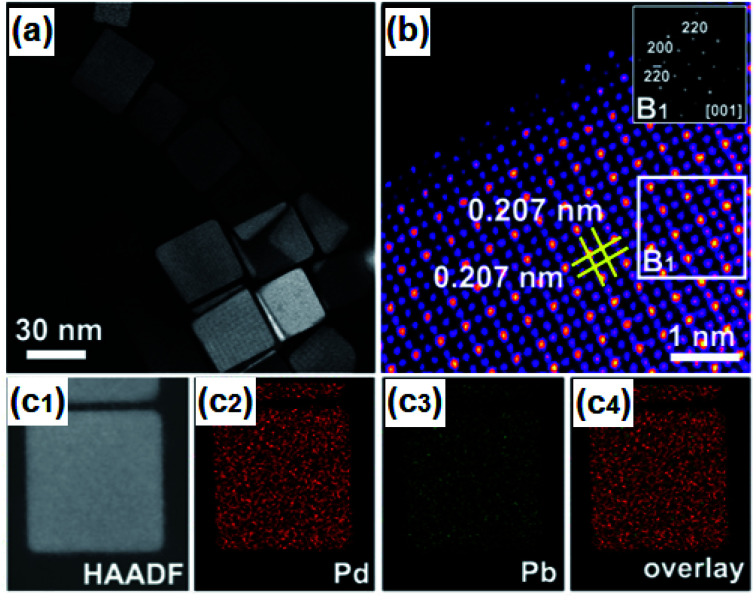
(a) HAADF-STEM image of Pd_3_Pb with (b) atomic-resolution HAADF-STEM image and FFT pattern in the inset, and (c1–c4) EDS mappings of Pd_3_Pb. Reprinted with permission from ref. 114, copyright 2019, American Chemical Society.

Taking advantage of this approach, nano particular PdBi_2_ ^119^ and Pd_3_Pb^114^ on Pt were prepared that showed the potential of nanoscale surface engineering by modifying rather inactive materials into highly efficient HER catalysts (Fig. 4). Both systems were attained at relatively mild conditions below 200 °C giving highly ordered NPs with improved intrinsic activity.^114,119^ Concurrently, IrNi and IrPt nanoframes were synthesized by reducing corresponding metal salt precursors and etching the framework. The as-attained IrNi systems encompassed a high ECSA and improved intrinsic activity compared to bare Ir. Not only was IrNi more active than bare Ir towards OER, but it also showed improved long-term stability mediating the challenging reaction in acidic media.^120^

At moderately high temperatures, we prepared the intermetallic FeSn_2_ phase by reducing a stirred mixture of iron(iii) and tin(ii) salt in a solution of polyethylene glycol and ethylene glycol under a rapid and constant flow of N_2_. The growth of intermetallic FeSn_2_ nanocrystals transpired by the interspersing of Fe into the interlayers of metallic Sn through a Kirkendall process (Fig. 3b).^102^ An analogous one-pot reduction was also used to prepare isotypic CoSn_2_ nanocrystals at 170 °C.^97^ Both materials were tested for water splitting catalysis with very high activity for both OER and HER.^97,102^

The hot injection is another viable reduction technique to synthesize monodisperse NPs, *via* rapid nucleation, followed by controlled growth of nuclei to form larger particles, and is well developed for the preparation of quantum dots. By varying stabilizing ligands, precursors, reaction time, and temperature, various phases of different sizes and morphologies can be achieved.^121^ In this respect, for the synthesis of popcorn-shaped GaPt_3_ NPs, Lim *et al.* used Ga(C_5_H_7_O_2_)_3_ precursor in oleylamine (OLA) and preheated to 200 °C under Ar atmosphere. To this solution, PtI_2_ was injected, and the mixture was heated up to 280 °C for 30 min followed by an instant cooling in a cold water bath. The intermetallic GaPt_3_ phase was examined for HER in all pH ranges (alkaline, acidic, and neutral).^122^ Alternatively, Lim *et al.* used the hot-injection method to prepare GaPd_2_ nanomaterials with different morphology such as polyhedrons, NPs, and nanowires just by using different solvents that exhibited both high catalytic HER activity and stability and were far superior to a commercial Pd catalyst.^123^ Furthermore, the same group synthesized GePt_3_ and Ge_2_Pt and concluded that by tuning the composition of the solvents and reagents as well as the reaction temperature, distinct phases of intermetallic compounds active for HER can be realized.^124^ In the meantime, Schaak and co-workers reported a lower-temperature route to colloidal metal silicide NPs such as Pd_2_Si, Ni_2_Si, and Cu_3_Si that were efficient for HER in acidic media.^125^

### 4.3 Hydro(solvo)thermal route

Hydrothermal or solvothermal synthesis is generally defined as a chemical reaction occurring at aqueous or non-aqueous solvents at elevated temperatures above the boiling point of the reaction medium and above atmospheric pressure. This synthetic technique is rather straightforward compared to the high-temperature solid-state and requires mostly moderate temperatures. By controlling the suitable selection of reaction conditions (precursor composition, pH, solvent, heating rate, temperature, pressure, concentration, *etc.*), pure phases with distinct sizes and morphology can be obtained. Currently, this method has been regarded as one of the versatile and relatively simple approaches to access numerous amorphous and crystalline structures.^81^

Although a variety of intermetallic compounds have been synthesized under such conditions and applied for manifold applications, their utilization as electrocatalysts for water splitting is rather limited.^126–129^ Recently, intermetallic NiMo with a surface containing SiO was obtained in reducing hydrothermally grown NiMoO_4_ in the H_2_ atmosphere. This porous NiMo composite was tested for HER with performances close to Pt.^130^ In another approach, Jin *et al.*, constructed intermetallic MoNi_4_ networks for OER, HER, and overall water splitting by treating nickel foam (NF) with ammonium molybdate *via* a hydrothermal reaction and then post-treated in H_2_ atmosphere at temperatures between 300–600 °C.^131^ Similarly, IrRu@Te intermetallic particles were attained when a mixture of Ir and RuCl_3_ was mixed together with Na_2_TeO_3_ in water and heated for 18 h in an autoclave at 180 °C. Without the addition of Na_2_TeO_3_, the unsupported IrRu phase was extracted but showed less activity when tested for OER under acidic and neutral conditions.^132^ Furthermore, PdCu_3_ was prepared solvothermally by mixing Pd(acac)_2_, Cu(acac)_2_ and cetyltrimethylammonium bromide (CTAB) together with OLA in a Teflon-lined autoclave at 180 °C for 24 h. The resulting Pd–Cu phase was successfully tested for HER with Pt-like activity.^129^

### 4.4 Wet impregnation

Wet impregnation is a well-known and effective technique for the preparation of heterogeneous catalysts, to significantly influence the physical and morphological properties by altering the microstructure.^133,134^ With suitable precursors, NPs are impregnated into a porous established electrode framework (support) to either enhance the electronic or ionic conductivity of the electrode or to enhance the catalytic activity, if both are not sufficient.^135,136^

Carbon-supported structurally ordered IrV_3_ NPs with a significantly reduced proportion of Ir and optimal distribution within the structure with a maximized number of neighboring Ir/V bimetallic sites were synthesized from a solution of IrCl_3_ and VCl_3_ mixed carbon black. To obtain the intermetallic phase, the well-dispersed suspension was dried and heated at 800 and 1000 °C under H_2_/Ar (5%) for 3 h. The ordered structure was able to efficiently promote the water dissociation and enhance the kinetics for alkaline HER.^137^ Likewise, PtNi/C was prepared in the same fashion but at a much lower temperature.^138^

The group of Li used N-doped graphite nanotubes as a highly active substrate for their intermetallic Ni–Mo nanocatalysts, (Fig. 3d). First, the Ni–N-doped graphite nanotubes were prepared by pyrolysis followed by acid etching, and then the N-doped graphite tubes with residue Ni (Ni-NGTs) were impregnated in an aqueous ammonium molybdate solution. Under reductive conditions at 600 °C, NGT supported Ni–Mo nanocatalyst was obtained with superior activity and stability in HER catalysis.^104^ Interestingly, recent work of Wu and coworkers revealed that highly efficient HER can also be accomplished by using MXene-supported Pt_3_Ti (Pt/Ti_3_C_2_T_*x*_) through wet-impregnation.^139^

### 4.5 Single source precursor-derived synthesis

The single-source precursor (SSP) approach is a versatile technique to explore bimetallic or even polymetallic materials. The pre-coordination in metalorganic precursors allows defined assembling of atoms on the molecular level and even altering the chemical behavior giving access to materials that are not approachable in traditional ways.^140^ SSPs enable a relatively good control over the composition, morphology, structural and electronic properties of desired materials. A large number of described metalorganic SSPs provide wide-range access to numerous materials and owing to their low decomposition temperature, various crystalline and amorphous structures have been reported.^1^

For intermetallic phases, Vela and co-workers compared the SSPs with the conventional precursor approach and they found that the SSP approach is more reliable as different reduction potentials of separate metal ions can be a limiting factor and lead to phase segregation or other unwanted side products.^141^ Further, the SSP techniques provide phase tuning by the change of ligand coordination environment and temperature that is generally lower than solid-state synthesis.^141,142^ Even though SSP is a very convenient strategy to synthesize phase pure intermetallic compounds for water splitting, less attention has been given considering the amount of complexities involved in designing a suitable molecular precursor.

Lately, DiSalvo's group synthesized an ammonium nickel molybdate with the formula NH_4_HNi_2_(OH)_2_(MoO_4_)_2_ and reduced the SSP to attain a Mo_7_Ni_7_ and a Ni_0.92_Mo_0.08_ phase. Thus yielded intermetallics exhibited promising results for alkaline HER.^143^ Learning lessons from our previous works,^19,26,28,144–148^ we reported a premediated synthesis of xanthene-based bis(germylene)-Ni complex and utilized it as a low-temperature precursor to form monodisperse ultra-small nickel germanide (NiGe) nanocrystals (Fig. 3e). Strikingly, the NiGe exhibited substantially low overpotentials for OER surpassing the state-of-the-art Ni-, Fe, Co, and benchmarked NiFe, and noble-metal-based catalysts.^105^ Furthermore, we were also able to access the intermetallic FeAs phase from a novel dinuclear arsenide iron complex L^B^FeAs_2_FeL^B^ (L^B^ = CH(C^*t*^BuNDipp)_2_) as SSP through a hot-injection method at moderate temperature. FeAs yielded in small spherical particles of about 10 nm in diameter with high surface area and was effective to catalyze OER.^149^ It is important to note that in recent years, SSP-derived materials have attained immense attention for the preparation of novel intermetallic compounds in nano form that otherwise are usually prepared *via* high-temperature routes and are relatively unexplored for electrocatalytic water-splitting.

### 4.6 Electrodeposition

A very common strategy to transform molecular precursors into electrocatalysts is the electrophoretic decomposition of these precursors at a certain applied potential to form a coating on a substrate.^1^ Though this strategy often lacks a complete understanding of the correlation between the electrochemical parameters and the resulting microstructure, it is a facile route to access amorphous and nanocrystalline structured films.^150^ Usually, intermetallic coatings protect against corrosion or improve mechanical properties and are of industrial importance.^151,152^

Pure intermetallic coatings fabricated by electrodeposition without using templates or post-heating techniques remain challenging and only a few examples exist.^153^ But the simplicity of this method and low cost in comparison to other deposition methods yielding in highly active species makes this approach attractive and admirable for application in electrocatalysis.^154^ Intermetallic Fe_4_Zn_9_–FeZn_13_ composite as reported by the group of Das or Ni–Zn coatings composed of NiZn, NiZn_3_, Ni_2_Zn_11_, and NiZn_7.33_ phases synthesized by Jovic *et al.* proved to be efficient for water electrolysis. The use of simple precursors such as sulfates or chlorides in an aqueous solution to achieve adherent and evenly distributed film at low temperatures makes this approach benign and feasible.^155,156^ On the other end, an approach described by Ballesteros *et al.* resulted in phase pure CuZn_5_ films on a Ni foil. Using an aqueous solution of copper and zinc sulfate glycine and a conventional three-electrode set-up, only the size of the spherical crystallites covering the electrode changed depending on the applied potential and the films tested for HER catalysis with moderate activity^154^ Alternatively, Jovic *et al.* synthesized phase pure NiSn films of varying morphology with varying the current density which showed remarkable activity for OER when tested in alkaline solution.^157^

### 4.7 Physical vapour deposition

Physical vapor deposition (PVD) is a widely known and applied technique for film preparation on various substrates based on evaporation or sputtering principles.^158^ This very energy-demanding technology allows precise regulation not only on morphology and layer thickness during film synthesis but also allows material composition far from the thermodynamic equilibrium while forming the targeted species permitting synthesis of ‘new’ materials.^158,159^

Utilizing this approach, Al–Ni electrodes were fabricated by PVD of aluminum onto a nickel substrate yielding in a coating ∼20 μm thick layers on the surface.^160^ The films were then annealed at 610 °C followed by selective aluminum leaching in a strongly alkaline electrolyte. XRD analysis revealed the formation of a mixture of the intermetallic Al_3_Ni_2_ and Al_3_Ni phases in the films since the Al_3_Ni_2_ phase grows faster than the Al_3_Ni phase. It was proposed that longer heat treatment will lead to solely an Al_3_Ni_2_ phase. The prepared electrodes were investigated for activity in alkaline water electrolysis with convincing results but showed severe instability of the materials.^160^

### 4.8 Other methods

**Fig. 5:**
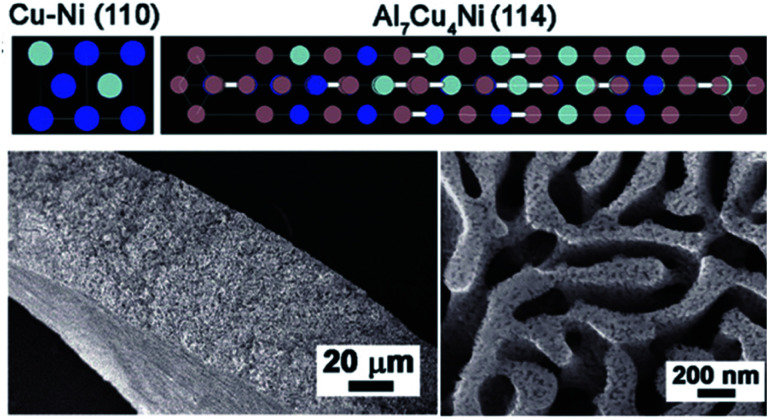
Top: Representative crystalline structure of Cu_4_Ni alloy and the Al_7_Cu_4_Ni intermetallic compound (Cu: blue; Ni: gray; Al: magenta). Bottom left: Cross-sectional SEM picture of Bi-NP Cu_12_Ni_1_Al_2.6_ Bottom right: SEM picture of top-view of Bi-NP Cu_12_Ni_1_Al_2.6_ with bimodal nanoporous structures consisting of micrometer- and nanometer-scale pore channels. Reprinted with permission from ref. 163, copyright 2018, Wiley-VCH.

Another convenient route to synthesize intermetallics is the melt-spinning technique. This method has shown great potential in nanostructuring to affect the physical and morphological properties of a material. Especially, for amorphous metallic compounds and due to their capacity of producing nanograins, melt-spinning has been utilized since the early 1980s.^161^ Several intermetallic phases such as FeCoNiAlTi HEI^162^ for HER and Cu/Al_7_Cu_4_Ni@Cu_4_Ni^163^ and NiIrRuAl NPNWs^164^ for overall water splitting have been fabricated *via* this facile approach to fine-tune their properties for efficient catalysis (Fig. 5).

One of the interesting approaches is high-intensity ultrasound treatment which has recently been used to synthesize the intermetallic AlNi phase. Here, commercially available Al–Ni alloy powder (50 wt% Ni) was dispersed in a water/ethanol mixture and sonicated for 1 h. The acoustic cavitation during the treatment is responsible for the initiation of redox processes on the particle surface leading to changes in its composition. Depending on the water: ethanol composition the different intermetallic Al_3_Ni_2_ and Al_3_Ni phases could be obtained and were successfully investigated for HER catalysis.^165,166^

Other ingenious methods such as laser ablation or plasma process synthesis have also been utilized to produce PtCo/CoO_*x*_ ^167^ nanocomposites and FeNi_3_ ^103^ for efficient OER. Lately, the ball milling technique was used to attain Ni_50_Mo_40_Ti_10_ and Ni_50_Mo_50_ ^168^ for HER and NiFe^169^ for efficient OER electrocatalysis. Based on the stoichiometry mixing the Fe and Ni at different molar ratios, FeNi_3_ nitrogen-doped carbon (FeN_3/_NC) was obtained using a plasma–liquid reaction (Fig. 3c). Plasma engineering improves the electrical properties and surface area and has proven to be a reliable electrocatalyst for OER and ORR.^103^

Apart from the synthesis, it is also important to thoroughly characterize the intermetallic compounds to understand their geometric and electronic properties so that they can readily be used for electrocatalysis. Usually, powder X-ray and single-crystal X-ray diffraction methods are used to determine their crystallinity, phase identification, and structural analysis with complementary theoretical calculations. Similarly, the surface structural characterization of (amorphous or crystalline) intermetallic compounds are performed by various techniques such as scanning electron microscopy, transmission electron microscopy, selected area electron diffraction, high angle annular dark-field scanning transmission electron microscopy, electron energy-loss spectroscopy, X-ray photoelectron spectroscopy. Besides, the coordination structure of intermetallics is revealed by Raman and X-ray absorption spectroscopy.^170^

## 5. Intermetallics for hydrogen evolution

Intermetallic phases have attracted many research groups to investigate their unique properties for water-splitting capability. Especially, intermetallic combinations of transition metals, as composite catalysts for HER, have been the subject of manifold experimental and theoretical studies.^171^ The pronounced synergism arises from a combination of transition metals with empty or half-filled d-orbitals with metals having more filled orbitals. The latter transition metals have internally paired d-electrons not available for bonding in the pure metal but in combination, giving exceptional charge-transfer capabilities to the formed intermetallic phase. This leads to exceptional electrocatalytic behavior in their intermetallic bi- or a multi-elemental compound that often surpasses precious metals as described by Brewer–Engel valence-bond theory for bonding in metals.^172–174^

According to the concept, various intermetallic combinations of transition metals, such as Hf_2_Fe, Hf_2_Co, PtMo, Pt–Mo, ZrPt, Nb–Pd systems, PdTa, and TiPt have been used as electrode material for HER in alkaline media and compared with the conventional transition metal-based materials. In contrast to the conventional cathodes, the intermetallics have shown significant electrocatalytic enhancement with Pt_2_Mo and Hf_2_Fe as the most efficient HER catalysts.^175,176^

### 5.1 Noble metal-based intermetallics

In electrocatalysis, especially, Pt-based intermetallics have shown high catalytic activity and often outperform non-noble-based alloys.^177^ Though in water-splitting, many transition metal-based electrodes have demonstrated high electrocatalytic performance in HER, Pt is still considered the highest active catalyst in alkaline and acidic electrolytes.^3^ With optimal structural properties to adsorb H^+^ more efficiently, it shows the lowest overpotential and is the most active material in HER.^178,179^ Therefore, attempts have been made to reduce the proportion of Pt in catalyst materials and design new materials by contemporaneously benefitting from cooperative effects in these composites and boosting electrocatalysis.^180^ Intermetallic phases of PtDy and PtMo_2_ with only 50 at% of Pt in their structure showed the highest activity in alkaline HER when compared to their corresponding phases with higher or lower Pt proportion.^107,181–183^ These observations have also been made for the Ho–Pt and Dy–Pt phases, which were significantly more active than the pure Pt during alkaline HER.^184^

Platinum–germanium NPs were synthesized using the hot injection technique to derive GePt_3_ and Ge_2_Pt, both tested for HER activity in 0.5 M H_2_SO_4_ electrolyte. While the Ge_2_Pt phase was found not to be active, GePt_3_ showed a low overpotential of *η*_–10_ = 53 mV and long-term stability during HER catalysis over 12 h. Compared to Pt black and Pt/C, GePt_3_ displayed higher specific activity corresponding to Pt content and higher mass activity compared to Pt black.^124^ The group of Tuan synthesized GaPt_3_ NPs in a similar approach to that of Ge–Pt that acted as highly efficient and stable electrocatalysts for HER. The catalyst was tested in 1 M KOH, phosphate-buffered solution (PBS), and 0.5 M H_2_SO_4_ electrolyte resulting in *η*_−10_ of 48, 103, and 27 mV, and operated stably under prolonged conditions for over 48 h.^122^ Another platinum-based intermetallic, Pt_3_Ti NPs on a two-dimensional transition metal carbide (Pt/Ti_3_C_2_T_*x*_) at different temperatures was investigated recently by Li *et al.* for HER in 0.1 M HClO_4_ as the electrolyte. The Pt_3_Ti NPs prepared at 550 °C (Pt/Ti_3_C_2_T_*x*_-550) showed the highest activity *η*_−10_ of only 32.7 mV.^139^

In the recent past, Chen *et al.* compared ordered and disordered phases of PtNi NPs for activity in alkaline HER. The unexpected results showed that the disordered phase was more active than the ordered one. Experimental and theoretical investigations suggested more Pt^IV^ and Ni^II^ species on the surface of D-PtNi/C as well as a synergetic effect between the formation of Ni/Pt–OH bonds and the increased disordering degree of Pt and Ni atoms on the surface of D-PtNi/C that enhanced the HER in comparison to the ordered species.^138^ Besides, Krstajic and co-workers synthesized MoPt_3_*via* an arc-melting route and investigated the kinetic and physical behavior of the material during acidic HER where they underlined a prominent role of Mo in the catalytic process once the MoPt_3_ electrode was activated by polishing the surface.^185^ In addition, a Pt-based silicide was prepared by magnetron-sputtering, giving an electrode of flat morphology containing uniformly spherical NPs with sizes of 10–20 nm. The Pt_2_Si was tested in 0.5 M H_2_SO_4_ electrolyte for HER resulting in *η*_−10_ of 78 mV and a Tafel slope of 30.5 mV dec^−1^.^186^

Electrode materials containing Pd have also been a focus of research as it is an interesting substitute for Pt. Although Pd is a noble metal, its price is much lower and shows similar activity to Pt in HER.^187,188^ Replacing the Pt with Pd has shown to be very efficient and phases of intermetallic PdBi_2_ has proven to be a sufficient replacement in HER catalysis and closely operating at Pt level.^119,189^ In a direct comparison of Pd-based intermetallic (Pd_2_Si) often show higher activity than their counterparts with non-noble transition metals (Ni_2_Si).^125^

Jana *et al.* synthesized PdCu_3_ by chemical reduction in OLA 180 °C and the addition of CTAB to control the reduction rates of Pd and Cu, giving spherical NPs that were highly active towards electrochemical HER. In acidic media (0.5 M H_2_SO_4_) high activity of PdCu_3_ was correlated to the dealloying of Cu from the structure resulting in the formation of active Pd sites with a low coordination number to facilitate the HER.^129^ Moreover, in a shape-controlled approach, gallium–palladium (GaPd_2_) nanomaterials were fabricated to drive the electrocatalytic HER. By changing the surfactant during synthesis, GaPd_2_ polyhedrons, NPs, and nanowires were achieved which were then examined for HER in 0.5 M H_2_SO_4_. The GaPd_2_ NPs exhibited the highest activity with *η*_−10_ of 24 mV and a Tafel slope of 57.2 mV dec^−1^ followed by the polyhedrons (*η*_−10_ of 33 mV) and the nanowires (*η*_−10_ 50 mV). The enhanced activity of the nanowires and stability for 24 h, superior to commercial Pd, was attributed to the increased surface area and the synergetic effect of Pd and Ga within the material.^123^ Lately, the group of Wu decorated intermetallic Pd_3_Pb nanoplates with a submonolayer of Pt to enhance acidic HER catalysis. They concluded that the very high activity resulted from the intermetallic substrate that stabilized the atomic structure of the active Pt layer as well as stabilized the electronic structure for effective electron transfer from Pd_3_Pb to Pt facilitating the electrocatalytic HER.^114^

Although Pt and Ru-based materials have been considered the best working electrocatalysts for HER, very recently, Ir has also garnered significant attention towards HER.^190,191^ As Ir can provide better stability at a high anodic potential than Ru in acidic conditions, it is considered as one of the very few catalysts driving efficient OER and HER in a broad range of pH.^191^ Making use of the bifunctionality with an oxophilic metal to promote the water dissociation and production of hydrogen intermediates, a structurally ordered intermetallic Ir_3_V was synthesized by Chen and co-workers to facilitate the HER. Vanadium being a highly oxophilic metal with the strongest metal–OH bond among the transition metals formed a structurally ordered phase with Ir (Ir_3_V) resulting in superior electrocatalytic behavior for alkaline HER. The achieved *η*_−10_ was only 9.0 mV even under prolonged conditions and revealed a Tafel slope of 24.1 mV dec^−1^ and clearly outmatching Pt/C and Ir/C references.^137^

### 5.2 Non-noble metal-based intermetallics

Completely replacing noble metals as catalyst materials remains a challenge in HER, especially since pure Mo, Fe, Co, or Ni bind H^+^ too weakly or too strong and therefore, are more or less inactive in HER.^192^ But when these metals were alloyed, a positive effect on the HER activity was observed with improved H^+^ adsorption characteristics and reduced overpotentials.^193^ Demonstrating exceptional physical and chemical properties, non-noble intermetallics have attracted many research groups to profoundly study this new class of materials for application in HER.^194^

In this respect, Rosalbinao *et al.* revealed that the electrocatalytic properties of Fe could be increased by forming intermetallic phases of Fe with rare earth metals. By the appropriate combination of 3d^6^-orbitals of Fe with 5d^1^-orbitals of Ce or La, according to the Brewer–Engel valence-bond theory, the HER performance of the final material was significantly enhanced to industrial standard. In their series, they found that the Fe–MM (MM = mixed metal) alloy, primary (Fe) crystals surrounded by a Fe_17_R_2_ phase (R content is Ce 5.5, Pr 1.5, Nd 1.5, Sm 0.5, Eu 0.2, Gd 0.3, Tb 0.5 and Dy 0.5 in at%) noted as Fe_90_MM_10_ and Fe_90_Ce_10_ to be most active for the alkaline HER.^89^ Furthermore, the positive effect of Ce in the structure was also observed in Ni–Zn-coatings containing the intermetallic NiZn_3_, Ni_2_Zn_11_, and NiZn_7.33_ phases. The composite material embedded with CeO_2_ NPs increasing HER activity than the one without the inclusion of CeO_2_.^156^

In order to surpass the performance of Raney nickel electrodes, Ti_2_Ni and TiNi were synthesized which showed considerably good performances in HER with small activation energies to mediate the reaction and extend higher stability than the Raney nickel references.^87,195,196^ Intense studies on the Ti–Ni system revealed a decreasing trend for HER activity in the order TiNi_2_ > TiNi_3_ > TiNi_4_ > Ti_2_Ni > TiNi > Ti_3_Ni > TiNi_0.7_ in which only TiNi_3_ exhibited the highest activity.^197^ The Raney nickel system with Ni_3_Al as analogous to TiAl with a similar face-centered cubic unit cell as well as structural order has also been investigated as the catalyst material for HER.^198^ Early reported phase pure Ni_2_Al_3_ and NiAl_3_ with minor impurities of Ni_2_Al_3_ were tested in 1 M KOH for HER activity and demonstrated good activity and stability. The porosity of the electrode material could be increased with Al content and the addition of Mo in the alloyed material could even further increase the HER activity.^92^ Comparable observations were made when the intermetallic phases of TiAl, FeAl, and NiAl were additionally alloyed with 2 at% of transition metals M = Ti, V, Cr, Mn, Fe, Co, Ni, and Cu. Additional alloying in general influenced activity, but, the more electron-rich the alloying metal became, the stronger the influence was on the overpotential and the HER activity.^199^ Similarly prepared Ni_3_Al by Wu *et al.* and Dong *et al.* through elemental powder reaction revealed enhanced porosity of the electrode materials and activity for alkaline HER. They observed that the intermetallic phase showed increased corrosion resistance and acquired a higher surface area due to the porous structure, as well as lower charge transfer resistance enhancing the HER compared to the pure Ni phase.^93,94^ Besides, Ni-based intermetallic phases combined with rare earth elements such as La or Mm (Mm = mixed metal) also displayed promotional effects on HER activity. Tamura's group studied LaNi_5_- and MmNi_5_-type alloys as electrode materials for HER and found that it shows Pt/Pd-like behavior and activity.^200–202^ Recently, a Ni–Co–Al lamellar nanostructure was attained by arc melting technique and tested for HER in a 1 M KOH electrolyte. Additional aging of Ni–Co–Al in an oven and etching in alkaline media, the material was dealloyed resulting in a higher ECSA resulting in a higher HER activity with *η*_−10_ of 178 mV when compared to a Ni foil standard.^111^ In a very different approach, Sun *et al.* synthesized Al_7_Cu_4_Ni@Cu_4_Ni core/shell nanocrystals by a melting-spinning method for highly efficient HER in alkaline media. The hybrid material showed *η*_−10_ of 139 mV and a Tafel slope of 110 mV dec^−1^ mediating the HER over a prolonged time of 8 h with a minimal shift of ∼14 mV. The high activity was accounted to the Ni incorporation that leads to a bimodal nanoporous architecture that simultaneously facilitates electrolyte access and electron transport as well as adapts the binding energy for H^+^ in nearby Cu atoms.^163^

Mo-based intermetallics are considered as a suitable substitute for electrode materials in HER since their activity often proved to be much higher with more favorable kinetics than pure Mo or Ni-based electrodes.^91,203–205^ McKone *et al.* directly compared the HER activity of Ni and Ni–Mo nanopowders revealing enhanced per-surface-atom activity compared to the bare Ni.^206^ Meanwhile, the group Jaramillo investigated intermetallic phases of NiMo, NiMoCo, CoMo, and NiMoFe for HER in acidic conditions and found a much higher activity than for Pt deposited on rotating disc electrodes.^207^ MoNi_4_ supported by MoO_2_ cuboids grown on NF *via* hydrothermal synthesis and annealing in the H_2_ atmosphere have shown remarkable activity towards HER, favoring the largely reduced Volmer step. With *η*_−10_ of 15 mV and a Tafel slope of 30 mV dec^−1^, this catalyst is considered as the best Pt-free HER catalyst in alkaline media.^208^ A study on ordered Mo_7_Ni_7_, disordered Ni_0.92_Mo_0.08_, and pure Ni powders for HER activity was also conducted that revealed the highest activity for the disordered species. Due to the high surface area, the disordered Ni_0.92_Mo_0.08_ favored the catalytic process, but when mass-specific activity was normalized to the surface area (determined by BET) rather than interfacial capacitance, the ordered Mo_7_Ni_7_ showed more intrinsically activity.^143^ A positive influence of Ti on the electrochemical performance in intermetallic NiMo was observed by Panek and co-workers. Ni_50_Mo_40_Ti_10_ demonstrated high intrinsic activity and much lower overpotentials for HER than the identically prepared Ni_50_Mo_50_.^168^ Enhancement of HER activity in Ni–Mo electrocatalysts was also achieved by including carbon nanostructures into the catalytic process. By caging Ni_4_Mo NPs into N-doped graphite tubes, a significant amplification of HER activity was observed with an *η*_−10_ as low as 65 mV a Tafel slope of 67 mV dec^−1^ to mediate acidic HER for 15 h.^104^ Additionally, the catalytic HER activity was improved by covering the surface of a porous NiMo network with N-doped graphene also strengthened the chemical stability of the system.^130^

Intermetallic stannides are known to possess unique chemical bonding with high electrical conductivity.^86^ The earlier work on nickel stannides, Ni_3_Sn and Ni_3_Sn_2_, fabricated *via* solid-state proved to be more active than their homometallic counterparts.^88,209^ Likewise, inspired by the previous works, Krstajic and his group electrodeposited Ni–Zn coatings and applied for alkaline HER and acquired promising results. The electrodes contained a mixture of different intermetallic phases (Ni, Ni_3_Sn, Ni_(1+*x*)_Sn_(0<*x*>0.5)_, Ni_3_Sn_4_) with varying chemical composition, phase composition, and morphology, and the effect of morphology on the activity was the most pronounced.^157^

Most recently, Suen and co-workers systematically synthesized a series of intermetallics, MCo_2_ (M = Ti, Zr, Hf, and Sc) using a rapid arc-melting method and studied their effects on HER. In this series, TiCo_2_ displayed a promising activity with an *η*_−10_ of 70 mV, a Tafel slope of 33 mV dec^−1^, and stability of 12 h and, this activity was comparable to a Pt/C standard.^109^

**Fig. 6:**
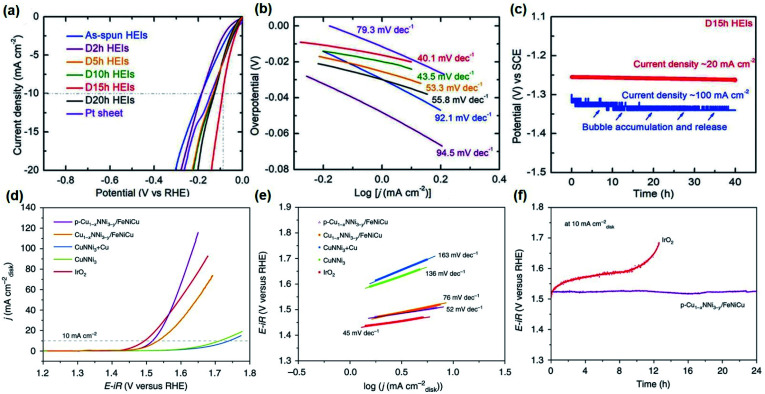
Electrocatalytic performance for HER and OER of selected catalysts: (a) HER LSV curves and (b) Tafel plots at a scan rate of 5 mV s^−1^ with *iR* loss correction of FeCoNiAlTi HEI and references in 1 M KOH. (c) Long-term stability measurement of the FeCoNiAlTi HEI at a current density of 20 and 100 mA cm^−2^ without *iR* loss correction. Figures (a–c) are reprinted with permission from ref. 162, copyright 2020, Wiley-VCH (d) OER LSV curves of Cu_1−*x*_NNi_3−*y*_/FeNiCu and references in 1 M KOH at 5 mV s^−1^. (e) Tafel slopes of Cu_1−*x*_NNi_3−*y*_/FeNiCu and references obtained from the steady-state measurements. (e) CP measurements of p-Cu_1−*x*_NNi_3−*y*_/FeNiCu and commercial IrO_2_ at 10 mA cm^−2^. Figures (d–f) are reprinted with permission from ref. 99, copyright 2020, Nature Publishing Group.

Efforts to stabilize and fine-tune the electronic structure by chemical synergism and structural site-isolation have been made to fabricate highly active non-noble catalysts. Based on the concept, FeCoNiAlTi, a high-entropy intermetallic (HEI) possessing the unusual periodically ordered structure was synthesized.^162^ The partially dealloyed HEI by acid etching showed high alkaline HER activity with an *η*_−10_ of 88.2 mV, a Tafel slope of 40.1 mV dec^−1^, and stability of 40 h (Fig. 6a–c).^162^ Here, the lowering of the overpotential was ascribed to the chemical complexity and unique atomic configurations that deliver a strong synergistic function to alter the electronic structure by optimizing the required energy barrier for hydrogen evolution.

Apart from conventional intermetallic compounds, sulfur-based intermetallic Al_25_Co_7_S_64_ was also prepared by dealloying Al_90_Co_10_ in 6 M KOH followed by solid-vapor sulfurization was highly active for HER in acidic electrolyte. The Al–CoS_2_ showed *η*_−10_ of 70 mV a Tafel slope of 153.5 mV dec^−1^ and was found to be stable for 12 h.^96^ An up-to-date table on the activity parameters of intermetallics has been provided in Table 1.

**Table 1:** Comparison of reported HER overpotentials of selected intermetallic catalysts at a given current densities in alkaline, acidic and neutral media^a^

Catalyst (substrate)	Electrolyte	Current density (mA cm^−2^)	Overpotential (mV)	Tafel slope (mV dec^−1^)	Reference
Al_7_Cu_4_Ni@Cu_4_Ni (direct)	0.1 M KOH	−10	139	110	163
CoSn_2_ (NF)	1 M KOH	−10	103	—	221
CoSn_2_ (FTO)	1 M KOH	−10	196	78	221
np-AlNiCoIrMo HEA (GC)	0.5 M H_2_SO_4_	−10	18.5	33.2	100
NiIrRuAl-3/1 (GC)	0.1 M HClO_4_	−10	14	23	164
D-PtNi/C (GC)	1 M KOH	−10	40	55	138
FeCoNiAlTi HEI (direct)	1 M KOH	−10	88.2	40.1	162
Fe_90_Y_10_ (direct)	1 M KOH	−250	470	125	89
Fe_90_MM_10_ (direct)	1 M KOH	−250	350	85	89
Ni–Zn (steel)	1 M NaOH	2.47	0	119	156
AlNiHT30 + L (Ni)	1 M KOH	−100	123	76	160
Ni_3_Al (direct)	6 M KOH	−90.1	426	219	93
Ni_2_Al_3_ @ 25 °C (Cu)	1 M KOH	−250	280	200	92
Ni_2_Al_5_ @ 25 °C (Cu)	1 M KOH	−250	253	157	92
NiAl_3_ @ 25 °C (Cu)	1 M KOH	−250	136	99	92
NiAl_3_Mo_0.153_ @ 25 °C (Cu)	1 M KOH	−250	57	134	92
NiAl_3_Mo_0.262_ @ 25 °C (Cu)	1 M KOH	−250	60	99	92
Ni–Co–Al (direct)	1 M KOH	−10	178	158	111
Ni–LaNi_5_ (Cu)	1 M NaOH	−250	330	101	222
Ni–MmNi_3.4_Co_0.8_Al_0.8_ (Cu)	1 M NaOH	−250	351	113	222
Mo_7_Ni_7_ (Ti)	1 M KOH	−3.7 (BET)	200	—	143
Ni_0.92_Mo_0.08_ (Ti)	1 M KOH	−1 (BET)	200	—	143
MoNi_4_ (Ni)	1 M KOH	−10	15	30	208
Porous MoNi_4_ networks (Ni)	1 M KOH	−10	28	36	131
Co_3_Mo (direct)	1 M KOH	−10	12	40	113
TiCo_2_ (direct)	1 M KOH	−10	70	33	109
HfCo_2_ (direct)	1 M KOH	−10	87	47	109
ScCo_2_ (direct)	1 M KOH	−10	87	36	109
GePt_3_ (FTO)	0.5 M H_2_SO_4_	−10	53	37	124
PdCu_3_ (GC)	0.5 M H_2_SO_4_	−10	50	34	129
Pt_2_Si (Ti)	0.5 M H_2_SO_4_	−40	78	30.5	186
Pt–Dy (direct)	8 M KOH (@25 °C)	−100	409	147	107
Pt–Dy (direct)	8 M KOH (@85 °C)	−100	139	141	107
GaPt_3_ (GC)	0.5 M H_2_SO_4_	−10	27	43.3	122
GaPt_3_ (GC)	1 M KOH	−10	48	63.1	122
GaPt_3_ (GC)	PBS	−10	103	85.3	122
PdBi_2_ (GC)	0.5 M HClO_4_	−10	78	63	119
GaPd_2_ nanowires (GC)	0.5 M H_2_SO_4_	−10	50	57.2	123
Pd_2_Si (Ti)	0.5 M H_2_SO_4_	192	192	131	125
Ni_2_Si (Ti)	0.5 M H_2_SO_4_	243	243	66	125
Ir_3_V (GC)	1 M KOH	−10	9.0	24.1	137
np-Co(Al) (direct)	0.5 M H_2_SO_4_	−10	239	153.50	96
Pt_3_Ti (carbon fiber)	0.1 M HClO_4_	−10	32.7	32.3	139
AL-Pt/Pd_3_Pb (GC)	0.5 M H_2_SO_4_	−10	14	18	114
NiMo-(a) (GC)	1 M H_2_SO_4_	−10	39	—	207
NiMo-(a) (GC)	1 M KOH	−10	30	—	207
Porous NiMo (GC)	0.5 M H_2_SO_4_	−10	22	37	130
NiMo-NHG (GC)	0.5 M H_2_SO_4_	−10	30	41	130
NiMo (1 mg cm^−2^) (Ti)	2 M KOH	−20	70	—	206
NiMo (3 mg cm^−2^) (Ti)	0.5 M H_2_SO_4_	−20	80	—	206
NiMo-NGTs (GC)	0.5 M H_2_SO_4_	−10	65	67	104
Ni_50_Mo_40_Ti_10_ (573 K) (direct)	5 M KOH	−100	215	249	168
Ni_50_Mo_50_ (573 K) (direct)	5 M KOH	−100	245	240	168

a FTO = fluorine doped tin oxide, GC = glassy carbon, NF = nickel foam.

Although Pt and Pt-based alloys remain as the most active materials for HER with *η*_−10_ between 10–20 mV and Tafel slopes ranging from 25–30 mV dec^−1^,^210^ recently, several non-noble metal catalyst based have attracted tremendous attention. Especially, Mo-based catalysts such as CoMoS_2_ with *η*_10_ = 56 mV and a Tafel slope = 32 mV dec^−1^ or Co–MoC with *η*_10_ = 46 mV and a Tafel slope = 46 mV dec^−1^ running stable for over 500 h, are considered potential candidates to replace Pt in electrochemical HER.^211,212^ In comparison, judging from the HER activities, the intermetallic-based catalysts have added advantages (see Section 8) and are on par with the other benchmarking systems, thus, are the most suitable candidates for HER catalysis.^213^

## 6. Intermetallics for oxygen evolution reaction

### 6.1 Noble metal-based intermetallics

Traditionally, RuO_*x*_ and IrO_*x*_ are still considered as the best working electrode materials for OER but their low abundance and high cost have limited their broad industrial application.^214,215^ However, the extraordinary stability especially in acidic media still makes these elements the first choice as anodes. Ruthenium catalysts usually are slightly more active and iridium-based catalysts are considered more stable for OER. Taking this into account, intermetallic catalysts based on Ru and Ir have been investigated both in acidic and neutral OER. Xu *et al.* reported IrRu@Te accessed *via* a solvothermal approach that exhibited low *η*_10_ of 220 (Tafel slope = 35 mV dec^−1^), 245, and 309 mV in 0.5 M H_2_SO_4_, 0.05 M H_2_SO_4_, and neutral electrolytes maintaining continuous OER catalysis in strong acid (0.5 M H_2_SO_4_) for 20 h at 10 mA cm^−2^ with a minimal decline in activity.^132^

Besides Ru and Ir, nanostructured Pt or prevailing intermetallic phases such as MoPt_3_–HfPd_3_ composite materials are proposed to provide substantially advanced electrocatalytic properties for OER.^216^ To develop corrosion-resistant catalysts for OER, Pt-based catalysts are the optimal alternative to realize highly active and long-term stable electrocatalysis. PtCo NPs embedded into CoO_*x*_ matrices showed remarkable resistance against aggregation and dissolution in alkaline media.^167^ The observed *η*_10_ of 380 mV (Tafel slope = 71.2 mV dec^−1^) for OER was far lower compared to Co-oxide and Pt/C with appreciated long-term stability.^167^ In a novel approach to assemble intermetallics, Lee and co-workers uncovered rhombic dodecahedral IrNi and PtNi nanoframes. Within them, IrNi exhibited surprisingly high activity towards OER catalysis with *η*_10_ = 325.8 mV a Tafel slope of 48.6 mV dec^−1^ and was structurally stable for 5000 cycles outperforming the Ir/C reference catalyst with a low Tafel slope of 48.6 mV dec^−1^. The enhancement in the activity of IrNi was ascribed to the unique structural and chemical composition of the material.^120^ Recently, Antonyshyn *et al.* synthesized the intermetallic Al_2_Pt phase for efficient OER giving rise to a moderate *η*_10_ of 450 mV that was better than the Pt reference. In addition to the activity, it showed a stable performance after activation during the first 100 h for more than 450 h.^108^

### 6.2 Non-noble metal-based intermetallics

In the last few years, numerous oxide-based materials have been investigated to address the concerns of the activity and stability of electrocatalysts during OER. Nevertheless, the lack of good electrical conductivity to enable an efficient charge transfer process constrains their widespread use. In this respect, transition metal-based intermetallics have been regarded as the most suitable electrocatalysts for OER as they comprise both desired chemical and physical properties. As intermetallics can also be cost-effectively prepared on a large scale, they have now gained enormous attention in the field of water-splitting catalysts.^217^ The group of Schuhmann recently investigated several intermetallics such as NiAs (*η*_10_ = 360 mV, Tafel slope = 58.7 mV dec^−1^) and Ni_2_Si (*η*_10_ = 410 mV, Tafel slope = 70.9 mV dec^−1^) for noble metal-free OER in alkaline electrolytes. They observed enhanced intrinsic OER activity in several metalloids resulting from unique electronic and structural properties in certain composite materials.^218^ Around the same time, Proost and co-workers studied a series of Ni–Al compounds for efficient water oxidation. They synthesized a series of pure Ni, AlNi_3_, AlNi, and a composite of AlNi_3_ and Al_3_Ni_5_ as electrode material on a Si substrate and compared their activity towards OER. Out of all, AlNi_3_ exhibited the highest activity with an *η*_10_ of 300 mV operating stably at this current density under prolonged conditions for 4 h and a Tafel slope of 103 mV dec^−1^.^219^ Additionally, annealing of as prepared Ni_3_Al at different temperatures to study the coarsening effect on HER activated the electrocatalyst. The most stable and active species was received by annealing deposited Ni_3_Al at 1160 °C.^95^ On the other hand, advanced stability, and high intrinsic activity were achieved by Yang's group using a eutectic high entropy alloy (EHEA) as a template to synthesize the multicomponent porous structure (MCPS) FeCoNiCrNb_0.5_ for OER catalysis. Their composite material showed excellent performance reaching 288 mV at *η*_10_ with a Tafel slope of 27.67 mV dec^−1^ and durability for 30 h.^101^

Of late, earth-abundant and considerably acid-stable tantalum-based intermetallics have also been considered as promising candidates for OER in acidic media. Schaak and co-workers reported a bimetallic series of Ta with Co, Ni, and Fe were produced by arc melting from the elemental metal powders in the stoichiometric composition. The obtained intermetallic Ni_2_Ta, Fe_2_Ta, and Co_2_Ta phases were pressed into pellets and used as electrodes for OER catalysis with Ni_2_Ta showing the highest activity at an *η*_10_ of 570 mV with the stability of over 60 h.^110^

Transition metal silicides have gained much attention lately for their specific crystal and electronic structures with high conductivity and stability that are the decisive factors for catalytic water-splitting.^220^ For application in anion exchange membrane fuel cells, bifunctional cobalt and nickel silicide have been decorated on silicon-oxy-carbide (SiOC) and tested for OER and oxygen reduction reaction (ORR). The synthesized Co/SiOC contained Co_2_Si and traces of pure Co while the Ni/SiOC consisted of a mixture of Ni_2_Si and Ni_3_Si. From both species, Ni/SiOC showed the highest performance in OER with *η*_10_ of 390 mV and exhibited better OER kinetics during catalysis.^223^ Following this, Kumar *et al.* used the molten LiI–KI eutectic mixture to access NiSi and Ni_2_Si *via* a reactive ionic Zintl solid (Na_4_Si_4_) and a Ni precursor. To reach a current density of 10 mA cm^−2^ an *η* of 570 mV was needed for NiSi and Ni_2_Si, which was comparable to a commercial IrO_2_ reference.^224^ In addition, porous Fe_3_Si, Fe_5_Si_3_, and FeSi have been prepared as intermetallic anode for zinc electrowinning. While the Fe_3_Si showed the best OER performance with *η*_10_ = 955 mV and a Tafel slope of 253 mV dec^−1^, the Fe_5_Si_3_ (*η*_10_ = 1072 mV, Tafel slope = 327 mV dec^−1^) exhibited better stability over 400 h.^90^

FeNi_3_ NPs on NC were synthesized by Mu and co-workers and the resulting FeNi_3_@NC electrocatalyst displayed excellent OER activity of *η*_10_ = 277 mV (Tafel slope = 77 mV dec^−1^) in alkaline conditions which they attribute to the unique structure with improved physical properties.^225^ To understand such systems more in-depth, Chen *et al.* studied a series of Fe–Ni intermetallics on NC gaining deeper insight into the role of iron in the structure. They synthesized various FeNi_3_/NC, Fe–FeNi_3_/NC, and Fe-enriched FeNi_3_/NC electrocatalysts and found the Fe-enriched FeNi_3_/NC was most active for OER with *η*_10_ of 360 mV and a Tafel slope of 82 mV dec^−1^ followed by Fe–FeNi_3_/NC (*η*_10_ = 390 mV, Tafel slope = 86 mV dec^−1^) and FeNi_3_/NC (*η*_10_ = 450 mV, Tafel slope = 141 mV dec^−1^) to reach the same current density concluding the higher Fe incorporation led to a positive effect giving more disorder to the structure.^103^ The influence of Fe in the Ni_3_Al structure was also investigated by Bai *et al.*, where the highest activity for OER was observed Ni_2/3_Fe_1/3_Al with an *η*_10_ of 299 mV and a Tafel slope of 58.9 mV dec^−1^, which was much lower compared to Ni_3/4_Fe_2/4_Al, Ni_1/2_Fe_1/1_Al, and other alloy materials.^169^

Das and co-workers prepared ultra-small intermetallic NiZn phase using Ni NPs with organometallic Zn precursor using low-temperature solution chemistry. This non-precious electrocatalyst showed a substantial OER in alkaline solution with an estimated *η*_10_ of 283 mV, a Tafel slope of 73 mV dec^−1^, and stability of 16 h and surpassed the activity of pure Ni and Ni_0.7_Zn_0.3_ alloy.^226^ Similar observations were drawn also in a comparative study on NiSn coatings on electrodes used for alkaline OER. The electrodeposited NiSn films at different potentials showed higher intrinsic activity than bare Ni films and the most active intermetallic phase NiSn100 reached *η*_40_ at 473 mV and a Tafel slope of 64 mV dec^−1^.^227^

Antiperovskite materials are intermetallic compounds with a perovskite crystal structure but instead, the anion and cation positions are interchanged in the unit cell.^228^ Transition metals and nitrogen or carbon can form antiperovskite structures at suitable compositions (AXM_3_; A = Cu, Al, Zn, *etc.*; X = N or C; M = Ni, Fe, Co, *etc.*), and can be well engineered to demonstrate versatile properties for OER catalysis.^99^ The p-Cu_1−*x*_NNi_3−*y*_/FeNiCu from Shao's group exhibited superior OER activity (*η*_10_ of 260 mV and Tafel slope of 52 mV dec^−1^) in alkaline media with at long-term stability over 24 h exceeding the activity of IrO_2_ (Fig. 6d–f).^99^ Inspired by this, Zou *et al.* developed a new intermetallic antiperovskite Co_3_InC_0.7_N_0.3_ that showed impressive OER activity with *η*_10_ of 260 mV, Tafel slope of 76.2 mV dec^−1^ and moderate stability of 10 h, and presented valuable insights into the active structure of this unique material class.^229^

We investigated a manganese gallide (MnGa_4_) and iron germanide (Fe_6_Ge_5_), both prepared by solid-state synthesis for potential application in alkaline OER. The MnGa_4_ is a d-sp bonded Hume–Rothery intermetallic compound with strong directional (covalent) bonds, metallic behavior, and antiferromagnetic ordering.^78,230^ The prepared MnGa_4_ has a defective CsCl structure, where three-fourths of the Cs atoms are eliminated to form corner-linked cubes (MnGa_8/2_) and proved to be a magnificent precatalyst for the electrochemical OER with a *η*_10_ of 293 mV (Tafel slope = 98 mV dec^−1^) for more than 24 h.^97^ Alternatively, Fe_6_Ge_5_ crystal structure consisted of a dense packaging of Fe and Ge atoms, built up by polyhedral of five different Fe atoms forming square pyramids, distorted octahedral and pentagonal prisms with the Ge atoms. In comparison to pure Fe-based materials, the Fe_6_Ge_5_ showed superior OER activity with *η*_10_ of 221 mV and a Tafel slope of 32 mV dec^−1^ and was constantly mediated the reaction under high potential for more than a day. In continuation, we further developed synthetic strategies to obtain NiGe^105^ and FeAs^149^ from molecular SSPs. The nanoparticular intermetallic phases proved to be very efficient during alkaline OER exhibiting low overpotentials and prolonged stability of three weeks.^105^ A chemical reduction approach was also utilized to derived phase pure FeSn_2_ nanocrystals recorded one of the lowest *η*_10_ of 197 mV (Tafel slope = 33 mV dec^−1^) with the stability of over 60 h. This system was found to be even superior to analogous Fe-, FeNi as well as noble-metal-based materials.^102^ An up-to-date table on the activity parameters of intermetallics has been provided in Table 2.

**Table 2:** Comparison of reported OER overpotentials of selected intermetallic catalysts at a given current densities in alkaline, acidic and neutral media^a^

Catalyst (substrate)	Electrolyte	Current density (mA cm^−2^)	Overpotential (mV)	Tafel slope (mV dec^−1^)	Reference
FeAs (NF)	1 M KOH	10	252	32	149
FeSn_2_ (NF)	1 M KOH	10	197	33	102
CoSn_2_ (NF)	1 M KOH	10	230	—	221
CoSn_2_ (FTO)	1 M KOH	10	299	89	221
np-AlNiCoIrMo HEA (GC)	0.5 M H_2_SO_4_	10	233	55.2	100
NiIrRuAl-1/3 (GC)	0.1 M HClO_4_	10	237	50	164
Fe_6_Ge_5_ (FTO)	1 M KOH	10	322	—	98
Fe_6_Ge_5_ (NF)	1 M KOH	10	228	56	98
NiGe (FTO)	1 M KOH	10	322	—	105
NiGe (NF)	1 M KOH	10	228	56	105
Porous MoNi_4_ networks (Ni)	1 M KOH	10	280	79	131
Co_3_Mo (direct)	1 M KOH	164	350	82	113
FeNi_3_@NC (GC)	1 M KOH	10	277	77	225
FeNi_3_/NC (GC)	0.1 M KOH	10	450	141	103
Fe-enriched FeNi_3_/NC (GC)	0.1 M KOH	10	360	82	103
MnGa_4_ (NF)	1 M KOH	10	293	98	97
Co/SiOC (GC)	0.1 M KOH	10	440	118	223
Ni/SiOC (GC)	0.1 M KOH	10	390	90	223
NiSi (GC)	0.1 M KOH	10	570	119	224
Ni_2_Si (GC)	0.1 M KOH	10	570	94	224
Ni_2_Ta (direct)	0.5 M H_2_SO_4_	10	570	—	110
Co_2_Ta (direct)	0.5 M H_2_SO_4_	10	600	—	110
Fe_2_Ta (direct)	0.5 M H_2_SO_4_	10	770	—	110
IrNi-RF (GC)	0.1 M HClO_4_	10	313.6	48.6	120
FeSi (direct)	1.6 M H_2_SO_4_	10	1107	634	90
Fe_5_Si_3_ (direct)	1.6 M H_2_SO_4_	10	1072	327	90
Fe_3_Si (direct)	1.6 M H_2_SO_4_	10	955	253	90
PtCo-1 (GC)	1 M KOH	10	380	71.2	167
PtCo-3 (GC)	1 M KOH	10	386	65.9	167
IrRu@Te (GC)	0.5 M H_2_SO_4_	10	220	35	132
IrRu@Te (GC)	0.05 M H_2_SO_4_	10	245	—	132
IrRu@Te (GC)	PBS	10	309	—	132
Al_2_Pt (direct)	0.1 M HClO_4_	10	450	—	108
AlNi_3_ (Si)	1 M KOH	10	300	46	219
AlNi_3_/Al_3_Ni_5_ (Si)	1 M KOH	10	390	52	219
AlNi (Si)	1 M KOH	10	310	47	219
Ni_3_Al N-0 (direct)	1 M KOH	10	320	112	95
Ni_3_Al N-1160 (direct)	1 M KOH	10	280	73	95
Ni_3_Al N-1200 (direct)	1 M KOH	10	293	85	95
Ni_3_Al N-1240 (direct)	1 M KOH	10	300	103	95
Ni_3_Al N-1280 (direct)	1 M KOH	10	305	108	95
Ni_2/3_Fe_1/3_Al (direct)	1 M KOH	10	299	58.9	169
Co_3_InC_0.7_N_0.3_ (GC)	1 M KOH	10	350	76	229
CuNNi_3_+Cu (GC)	1 M KOH	10	510	163	99
CuNNi_3_ (GC)	1 M KOH	10	480	136	99
Cu_1−*x*_NNi_3−*y*_/FeNiCu (GC)	1 M KOH	10	300	76	99
p-Cu_1−*x*_NNi_3−*y*_/FeNiCu (GC)	1 M KOH	10	280	52	99
NiZn (GC)	1 M KOH	10	283	73	226
Cu_s_Sn@Cu (Cu)	1 M KOH	50	384	177	232
NiSn10 (Ni)	1 M NaOH	40	541	60	227
NiSn60 (Ni)	1 M NaOH	40	516	63	227
NiSn100 (Ni)	1 M NaOH	40	473	57	227
FeCoNiCrNb_0.5_ (direct)	0.1 M KOH	10	288	27.67	101
AlNiHT10 + L (Ni)	1 M KOH	100	338	—	160

a FTO = fluorine doped tin oxide, GC = glassy carbon, NF = nickel foam.

The most active non-noble benchmarking systems for OER are based on metal oxides, (oxy)hydroxides, and chalcogenides. For instance, the *η*_10_ of several present NiFe or NiCo (oxy)hydroxides and oxide systems range only between 180 to 240 mV with Tafel slopes of 28–35 mV dec^−1^ that also remain stable for several hours even at higher current densities of 500 mA cm^−2^ and 1000 mA cm^−2^.^33^ Similarly, most of the other active materials such as perovskites, phosphides, alloys and carbides displaying overpotentials between 200–300 mV with Tafel slopes ranging from 40–80 mV dec^−1^ to facilitate OER have lately been reported. Interestingly, the OER activities of these materials are matched closely with recently reported intermetallic phases and likely to improve significantly based on the structural tuning, composition, and electronic conductivity (see Section 8) making them an intriguing class of materials for further investigations.^231^

## 7. Intermetallics for overall water-splitting

Electrocatalytic overall water-splitting with intermetallic phases is a relatively nascent field with only a few reported examples.^29,30^ An early study on several alloys and intermetallics, *e.g.* Ti_2_Ni, TiNi, TiCu, TiCo, and ZrNi from Miles revealed that proper combinations of elements can be favorably modified to facilitate HER and OER.

Although no further investigations on structure, morphology, or physical properties were carried out, a correlation on periodic trends of overpotential and atomic number with modern values for electronic work functions was derived for water splitting.^87^ To increase the roughness factor, Møller's group fabricated AlNi electrodes for alkaline water electrolysis and highlighted the importance of large ESCA and low charge transfer resistance for efficient water splitting, although, the electrodes deteriorated dramatically during the harsh reaction conditions.^160^ Taking inspiration from the previous works, we carefully designed and developed a novel strategy for a controllable synthesis CoSn_2_ nanocrystals that contained both active and conducting sites.^221^ As anticipated, CoSn_2_ displayed an excellent *η*_10_ of 230 mV for OER, *η*_−10_ of 103 mV, and necessitated a cell potential of 1.55 V to reach 10 mA cm^−2^ in alkaline solution with almost 100% FE surpassing their Co and Sn counterparts.

**Fig. 7:**
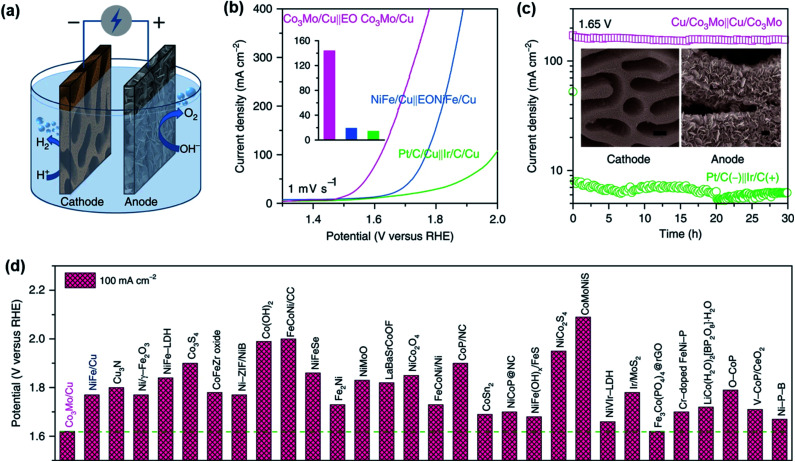
(a) Illustrated set-up of an overall water splitting cell with Co_3_Mo/Cu and EO Co_3_Mo/Cu electrodes as cathode and anode. (b) LSV curves for overall water splitting of Co_3_Mo/Cu‖EO Co_3_Mo/Cu and references in a 1 M KOH aqueous electrolyte and 0.5 M NaCl (Inset: comparison of the received current density at a cell potential of 1.65 V). (c) Long term measurement of Co_3_Mo/Cu‖EO Co_3_Mo/Cu at 1.65 V in comparison to Pt/C/Cu‖Ir/C/Cu with inset depicting SEM images of Co_3_Mo/Cu cathode and EO Co_3_Mo/Cu anode after prolonged electrocatalysis (scale bar = 400 nm). (d) The activity comparison of the cell potential of Co_3_Mo/Cu in alkaline media at a current density of 100 mA cm^−2^ with various reported. Reprinted with permission from ref. 113, copyright 2020, Nature Publishing Group.

In search of new overall water splitting electrocatalysts, Zhang and co-workers synthesized a noble metal-based intermetallic NiIrRuAl-1/3 with low content of Ir and Ru (34 at%) to form a hierarchically nanoporous nanowire structure with high ECSA that showed *η*_10_ of 237 mV (Tafel slope = 50 mV dec^−1^) for OER, while the NiIrRuAl-3/1 performed best in HER with *η*_−10_ of 14 mV (Tafel slope = 23 mV dec^−1^) and a cell potential of 1.464 V was required for NiIrRuAl-1/3//NiIrRuAl-3/1 in an acidic media with extended stability of 35 h outperforming IrO_2_ and Pt/C references.^164^ At the same time, a three-dimensional porous intermetallic MoNi_4_ network was constructed by Jin *et al.* that demonstrated impressive performance as electrode material displaying *η*_−10_ of the only 28 mV (Tafel slope = 36 mV dec^−1^) for HER, *η*_10_ of 280 mV (Tafel slope = 79 mV dec^−1^) for OER. When examined for bifunctional overall water splitting, a cell potential of 1.58 V at a current density of 10 mA cm^−2^ was observed with over 24 h stability, which was significantly better than the reference Ni electrodes.^131^ Soon after, an Al_3_(NiCoIrMo) intermetallic phase was accessed *via* solid-state route and partial dealloying to a reduced content of Ir in the structure (∼20 at%). The quinary nanoporous np-AlNiCoIrMo HEA needed *η*_−10_ of 18.5 mV (Tafel slope = 33.2 mV dec^−1^) for HER, *η*_10_ of 233 mV (Tafel slope = 55.2 mV dec^−1^) for OER, and overall cell voltage of 1.505 V with high durability of 48 h.^100^ Moreover, Shi and co-workers developed self-supported monolithic nanoporous Co_3_Mo/Cu electrodes from Cu_12−*x*−*y*_Co_*x*_Mo_*y*_Al_88_ precursors, which showed unexpected performance for HER with *η*_−10_ of merely 12 mV (Tafel slope = 40 mV dec^−1^) while *η*_164_ for OER was 350 mV (Tafel slope = 82 mV dec^−1^) (Fig. 7).^113^ The catalyst was further tested for overall water-splitting resulting in a cell potential of 1.65 V to produce a current density of 100 mA cm^−2^.

It should be noted here that the activity presented in Tables 1 and 2, are only based on the overpotential, Tafel slope, electrolyte, and substrate effects. As the experimental conditions (pH, temperature, loading amount, film thickness, *iR* compensation, *etc.*) can differ in each case, the reader is referred to the respective references.

## 8. Insights into the active structures

**Fig. 8:**
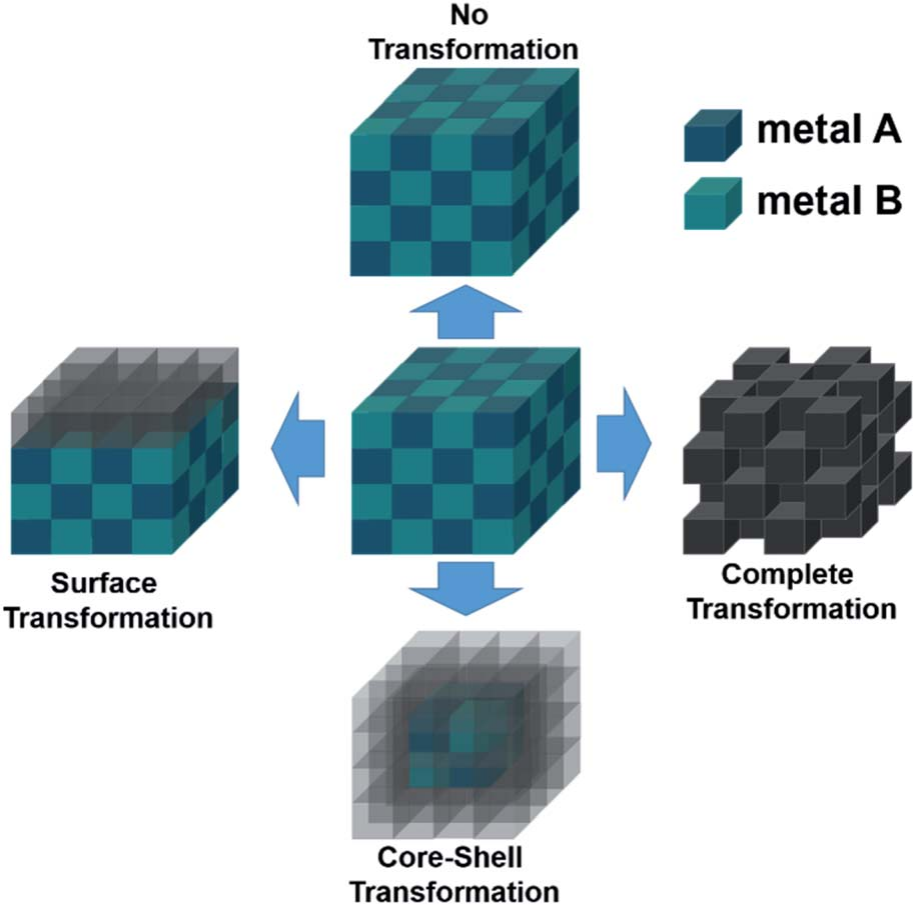
Possible transformation pathways of a precatalyst into an active catalyst during catalytic OER and HER conditions.

Intermetallics are considered as a highly stable compound with higher resistance against chemical oxidation than single metal compounds due to their ordered structure with d-orbital interaction and covalent bonding.^30,233^ Currently, the high activity of intermetallic phases in the two different half-reactions of water splitting is often ascribed to the pronounced synergism of the different metallic species (active or electrically conductive), when no change in the original or pristine state of the structure was observed under catalytic conditions.^96,171,229^ If the catalyst is not stable during OER or HER catalysis, then it will be in its precatalytic state and the so-called pre-catalyst can transform in three possible ways: (i) near-surface, (ii) partial (core–shell), and (iii) complete transformation resulting into crystalline or amorphous structures (Fig. 8).^16,234^ In the case of near-surface, only the outermost layer of the catalysts are transformed typically by leaching or oxidation so that the reactants can participate in the reaction and contribute towards the net catalytic activity. The extent of transformation mainly depends upon the porosity or voids created by leaching (non)metals. In many cases, the structural transformation of the precatalyst is not continuous and ceased after a certain time interval when electrolyte can no longer penetrate through the precatalyst's core forming a stable core–shell phase. Under prolonged electrolysis, this process encompasses deep inside the core of the particles finally forming a stable active structure. The degree of conversion is not only limited to the leaching phenomenon but also depends on the size, morphology, composition of particles as well as the complex bonding situations of intermetallics. The transformation primarily results in increased ECSA, enhanced defect/disordered sites, higher electrical conductivity, surface or bulk activity, and structural porosity. In the following, we describe the type of transformation attained for intermetallic phases under electrocatalytic HER and OER conditions.

### 8.1 Active sites in HER

**Fig. 9:**
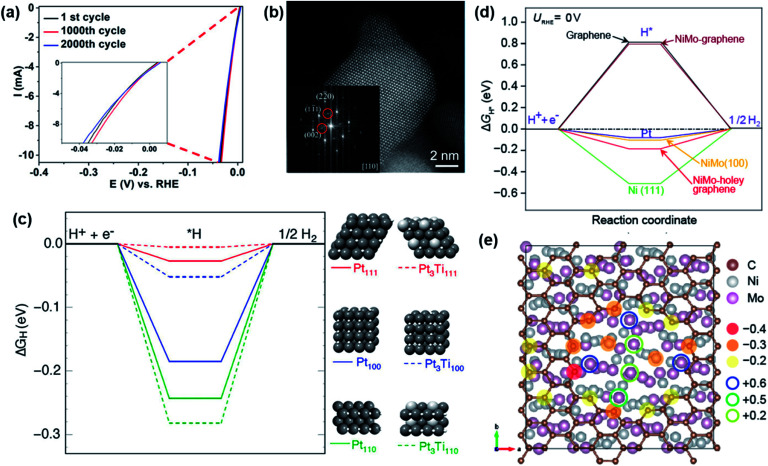
(a) HER LSV curves before and after the stability tests of intermetallic Pt_3_Ti. (b) HAADF-STEM image of Pt/Ti_3_C_2_T_*x*_-550 (after 1000 cycles in HER) where the inset shows FFT pattern of a Pt_3_Ti NP. (c) DFT calculations of the free energy diagrams of the hydrogen evolution at the surfaces of Pt (111), Pt_3_Ti (111), Pt (100), Pt_3_Ti (100), Pt (110), and Pt_3_Ti (110). Figures (a–c) are reprinted with permission from ref. 139, copyright 2019, American Chemical Society (d) Δ*G* profiles calculated by DFT for the NiMo composites, showing the calculated free energy diagram of HER at the equilibrium potential for Pt, Ni(111), NiMo(100) graphene covered and bare as well as graphene covered and with a ∼1 nm hole, and a graphene sheet. (e) Bader charge population analysis of graphene covered NiMo(100) with a ∼1 nm hole. Figures (d and e) are reprinted with permission from ref. 130, copyright 2018, American Chemical Society.

Most of the reported intermetallic compounds have shown no indication of transformation acting as chemically stable and highly resistant in a harsh HER environment.^234^ The high activity is explained by the specific surface composition of the formed materials.^88^ For instance, the remarkable HER performance of Ni and rare-earth-based intermetallics (Ni–LaNi_5_ and Ni–MmNi_3.4_–Co_0.8_Al_0.8_) was discussed in light of Brewer–Engel valence bond theory implying a synergistic effect of the hyper-electronic character of the Ni and the hypo-electronic character of the rare-earth element on the electrode surface to facilitate H^+^ adsorption (Heyrovsky step).^222^ The same synergistic effect of intermetallics was also revealed for Ni_4_Mo and Ni_3_Mo where d-orbitals of nickel (group VII) are more filled than those of molybdenum (group VI). Upon alloying, the d-orbitals interact and the catalyst becomes more active for HER when the d-band vacancies are introduced and becomes less active as d-band vacancies are filled.^91^ Similar observations have also been made for PtNi, LaNi_5_, TiNi_3_, ZrNi_3_, HfPd_3_, MoPt_3_, FeR (R = rare earth metals), and Ni–Mo-NGTs.^89,104,138,176,222^ In the case of NiIrRuAl, the charge transfer induced Ni gave rise to modified surface valence states that optimized the H^+^ adsorption and improved the catalytic HER activity.^164^ Pt_2_Si showed higher catalytic HER activity than Pt, by facilitating HO–H bond breaking and H^+^ adsorption through the incorporated Si and remained unchanged in the catalytic process.^186^ In addition, Pt_3_Ti^139^ and TiCo_2_ (ref. 109) also showed ideal conditions to adsorb H^+^ on the surface. Besides the optimal adsorption of H^+^ (Fig. 9c), the Pt_3_Ti proved to be very stable under electrocatalytic conditions in acidic HER. Only a minor change in activity was measured after 2000 CV cycles and the TEM showed that Pt_3_Ti phase was still intact (Fig. 9a and b).^139^

Along with the synergistic effect to enhance structural and electronic properties, and enhancement of the mass-specific surface area in the intermetallic phases has been observed, which significantly contributes to the HER activity.^124,137,143^ NiMo exhibited a much higher surface area and a larger turnover frequency per-surface atom resulting in a higher mass activity for HER. The tested intermetallic phase was stable in alkaline media and only degraded in acidic media. By the calculations of DFT and Bader charge population, it was demonstrated that NiMo had its optimal adsorption conditions and was stable when NiMo was covered with holey graphene and therefore possessed more active sites (Fig. 9d and e).^206^ By additionally covering the surface of NiMo with graphene, the dissolution of the intermetallic phase in acidic media was drastically reduced and enhanced the catalytic activity of the material.^130^ Ni_50_Mo_40_Ti_10_ and Ni_50_Mo_50_ were also found to be stable and highly active due to their enlarged surface area. Additionally, it was found that the presence of Ti in NiMo further increased the intrinsic activity for HER.^168^ The comparably high surface area that maximizes the exposure of active sites was also observed in Ti–Ni,^196^ Fe–Zn^155^ Pd_2_Si,^125^ Ni_2_Si,^125^ GaPt_3_,^122^ GaPd_2_,^123^ and Pt–Dy^107^ resulting in high HER performance. Jin *et al.* ascribed their efficiency and durability for their np-AlNiCoIrMo HEA to the high-entropy stabilizing effect of the quinary compound. The five different uniformly distributed elements with unique physiochemical properties attributed synergistically to the formation of the porous structure that was retained during HER.^100^ Similar conclusions were drawn for the FeCoNiAlTi HEI.^162^ Intriguingly, the bifunctional Co_3_Mo/Cu exhibited efficient HER and remained unchanged. The high activity of Co_3_Mo was attributed to the surfaces precisely composed of Co and Mo atoms, wherein their dissimilarity results in both ligand effect and strain effect to regulate the adsorption energies of the H* intermediate, leading to a high performance HER electrode.^113^

Apart from electrochemically stable electrodes, many reports show surface passivation and even transformation of the precatalyst during HER. In such cases, the transformation of the surface of the film or particles leads to a core–shell structure and the active materials typically exhibit an increased surface area.^234^ Despite reports on electrochemically stable Ni_3_Al electrodes for HER, Al-based electrodes tend to transform during catalysis due to Al leaching.^93,94,235^ Highly porous Ni_3_Al from Liu's group showed slight surface passivation after strongly alkaline (6 M KOH) HER catalysis. It was expected to form NiO and NiOOH/OOH on the surface, but it is unclear whether the formed species influenced the HER activity.^93^ In other reports, the Al leaching in alkaline solution is intended to increase the electrode activity by increasing the surface (porosity effect) through the created voids, and some phases were not stable and disintegrate during leaching.^92^ However, the intensity of the leaching process was dependent strongly on the initial stoichiometric and elemental composition, the structure, and the morphology of the starting material as well as the electrolyte.^234^ A loss of Al was also confirmed for Al_7_Cu_4_Ni@Cu_4_Ni in alkaline HER forming a core–shell structure. The stability of the material was ascribed to the stable Cu_4_Ni shell, in which the Ni atoms anchored with subsurface Al atoms forming strong Ni–Al bonds and suppress diffusion of Cu atoms.^163^ The same results were obtained for PdCu_3_ and PdBi_2_ where a loss of Cu and Bi resulted under HER forming *in situ* a Pd rich catalyst. Long-term HER experiments confirmed that the shell of the catalyst inhibited further leaching and remained stable once formed that indeed increased the H^+^ adsorption as well as the charge transfer through the catalyst.^119,129^ Analogous HER reaction pathways were observed when we investigated CoSn_2_ as a catalyst for water splitting. The surface leaching of Sn from the outermost layer of CoSn_2_ exposed abundant Co^0^ sites for HER for facile H^+^ adsorption and reduction to form H_2_ while unchanged bulk phase contributed in terms of electronic conductivity.^221^

A more drastic transformation of an intermetallic phase into an oxide phase during HER was observed in Ni–Co–Al on a Ni-foil by Zhou *et al.* X-ray photoelectron spectroscopy (XPS) data clearly revealed, that after HER, Al was oxidized into Al_2_O_3_; Ni into NiO and Al_2_NiO_4_ and Co into Co_3_O_4_ and Al_2_CoO_4_. Since no oxide species was present on the precatalyst material, the Al_2_NiO_4_/Al_2_CoO_4_ was considered as an active species contributing to the electrocatalytic performance towards HER in alkaline solution.^111^

### 8.2 Active sites in OER

In stark contrast to HER catalysts, the strongly oxidizing conditions of the OER force most of the non-oxides inexorably to form oxides and (oxy)hydroxides (at least at the surface) due to their thermodynamically more favorable stability.^236^ However, some of the reported intermetallic compounds show almost no or limited change in their crystal structure and morphology under OER conditions.^234^ For example, Mukherjee and co-workers reported intermetallic PtCo with CoO_*x*_ composite for efficient OER and ORR. The sponge-shaped CoO_*x*_ matrix surrounding PtCo NPs enforced synergic “spillover” effects and helped to preserve the structural and morphological integrity of PtCo NPs preventing their aggregation and dissolution in alkaline electrolytes.^167^ Likewise, the microstructure and element distribution of the np-AlNiCoIrMo HEA revealed no obvious microstructure coarsening and uniformly element distribution without aggregation after the OER durability test. It was stated that the bicontinuous nanoscale ligament-pore structure facilitates mass transport (both electrolyte and gas) and provided a conductive network for fast electron transfer and efficient electrochemical reactions.^100^ No visible transformation was reported for the intermetallic Ni/SiOC phase even after 10 000 CV cycles of OER which was attributed to the presence of Ni^III^, giving increased conductivity to the material and providing higher affinity towards adsorption of OH* anions enhancing the OER.^223^ Retention of structural stability after OER was also reported for intermetallic AlNi_3_, AlNi_3_/Al_3_Ni_5,_ and AlNi phases, however, a minor amount of Al leaching was unavoidable.^169^

As discussed earlier, in most cases, the transformation of the (pre)catalyst into the active species during OER catalysis is imminent. The nature of the formed catalyst is mainly defined by the structure of the precatalyst, electronic properties, the leaching ability and size of the leaching ion, and the transformation conditions strongly influenced by the pH of the electrolyte as well as the applied potential.^16^ The intermetallic NiSn synthesized by Jović *et al.* slowly oxidized on the surface into a β-NiOOH in alkaline OER that γ-NiOOH activated from bare Ni.^227^ Using Raman spectroscopy, Shen's group was also able to uncover *in situ* NiOOH active layer by etching of MoNi_4_ in alkaline OER conditions.^131^ The core–shell Cu_1−*x*_NNi_3−*y*_/FeNiCu, reported by Shao's group described the FeNiCu-(oxy)hydroxide as the active species in the HER process, that retained structural integrity post 24 h of OER.^99^ The group of Grin observed that the bulk Hf_2_B_2_Ir_5_ and major volume close to the surface did not change under the OER conditions. The formation of monoclinic HfO_2_ and IrO_*x*_(OH)_*y*_ (SO_4_)_z_ particles upon anodic treatment of the Hf_2_B_2_Ir_5_ was mainly related to the oxidation of the secondary phase HfB_4_Ir_3_ that was also present in minor amounts in addition to the self-controlled near-surface oxidation of Hf_2_B_2_Ir_5_. They concluded that the chemical bonding features of the Hf_2_B_2_Ir_5_ compound with a cage-like Ir–B framework, hosting Hf atoms, only allow near-surface oxidation and inhibit deep Ir leaching.^112^ Zhang and co-workers showed that the surface oxidation of NiIrRuAl led to an improvement of catalytic activity. By tuning the atomic ratio of Ir/Ru, it was demonstrated that a higher Ru content improves the intrinsic catalytic activity while more Ir effectively generates Ir oxides on the surface to retain the catalytic stability. More Ni content weakened the adsorption of oxygen-based intermediates by shifting down the Ir d-band center signifying its role in OER activity and it also leached from the surface structure leading to an increased concentration of oxygen species, drastically enhancing the OER activity.^164^

The persistent surface oxidation of intermetallics under OER often leads to a core–shell structure that usually consists of a crystalline or amorphously active shell and a conductive core that supports cooperative interactions of the elements.^234^ While thickness and properties of the surrounding layer vary depending on the starting material and transformation conditions, it is often observed that the oxide coating actively inhibits further oxidation of the material underneath.^234,236^ As an example, Ni_2_Ta, Co_2_Ta, and Fe_2_Ta anodes slowly corroded under acidic OER conditions and formed a protective oxide layer that indeed acted as a passivating oxide coating, allowing subsurface atoms to continue to participate in OER while limiting their dissolution.^110^ In another example, acid etching of IrNi to obtain nanocages induced an IrO_*x*_ layer on the surface. This layer increased under continuous OER operation resulting in core–shell IrNi@IrO_*x*_. The unique feature of the inner lying IrNi and outer IrO_*x*_ phase was concluded to be responsible for the high OER activity.^120^ In a recent approach, we observed similar transformations in FeSn_2_. After alkaline OER, the FeSn_2_ generated a goethite α-FeOOH shell with FeSn_2_ core. The α-FeOOH shell facilitated the OER while the intact intermetallic core enhanced electrical conductivity.^102^ Besides, the Ni_2_Si and NiSi prepared by Kumar *et al.* suffered substantial reorganization of the structure for alkaline OER. The SiO_*x*_ species leached from the surface continuously to form an active NiOOH shell on both NiSi or Ni_2_Si.^224^

**Fig. 10:**
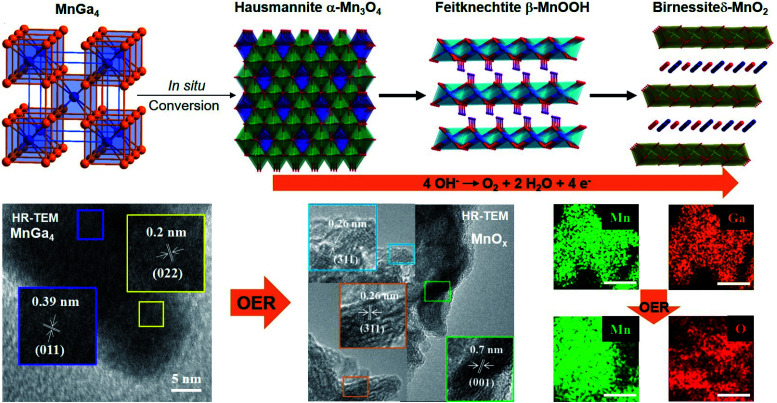
Proposed mechanism for the electroconversion of MnGa_4_ to δ-MnO_2_ with its intermediates (top). TEM images of MnGa_4_ and the transformed oxide species after OER in alkaline media (bottom left). EDX mapping of Mn and Ga in MnGa_4_ before alkaline OER and Mn and O mapping after. Adapted with permission from ref. 97, copyright 2019, Wiley-VCH.

In many cases, the complete degradation of the precatalyst into a more crystalline or amorphous (oxy)hydroxide under OER has been reported. As the material is oxidized, it undergoes significant leaching of the nonmetallic component and facilitates major structural reorganization with substantially enhanced ECSA from the newly porous bulk-active structures that in turn increases the number of active sites for the OER.^234^ Different observations were made by Møller's group for their Al–Ni phase where they postulated that in alkaline conditions the material is thermodynamically not stable at zero potential and undergoes corrosion. Another possibility for dissolution was localized acid formation inside the porous structure during OER resulting from disturbances of oxygen bubbles on OH^−^ migration into the inner active sites of the catalyst. As a consequence the pH is locally decreased, hence the structure is further weakened and corrosion will ultimately severely damage the structure.^160^ Similar observations were also made for intermetallic MnGa_4_ where three distinct crystalline MnO_*x*_ phases: birnessite δ-MnO_2_, feitknechtite β-MnOOH, and hausmannite α-Mn_3_O_4_ were confirmed after the alkaline OER that was accompanied by an almost complete Ga loss from the structure (Fig. 10).^97^ The transformation trend was also extended for SSP-derived FeAs and NiGe precatalysts. In the case of FeAs, an active 2-line ferrihydrite phase was formed after alkaline OER with the severe dissolution of As in the structure. The Raman and *in situ* XAS described the reaction pathway and the defective edges/sites, as well as the presence of surface tetrahedral coordinated Fe^III^ atoms, were ought to be the active centers for OER catalysis.^149^ On the other end, NiGe produced γ-NiOOH. The activity was attributed to the structural flexibility of Ni sites triggered by the defected structure with ionic intercalation of OH^−^/CO_3_^2−^ between the large interplanar spacing of γ-NiOOH, In both cases, the transformation resulted in higher ECSA values, and better electronic conductivities to promote favorable OER kinetics with improved charge transfer properties to facilitate the OER.^105^ Of late, we reported an intermetallic Fe_6_Ge_5_ as a novel alkaline OER precatalyst forming first an *in situ* metastable core–shell structure that slowly collapses in prolonged OER conditions finally to form a Fe^III^O_*x*_H_*y*_.^98^

**Fig. 11:**
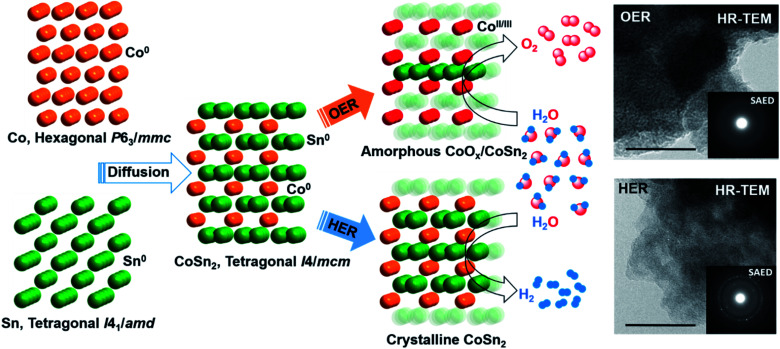
A synthetic approach to nanostructured CoSn_2_, with structural modification during alkaline HER and OER (left). TEM images of showing amorphous CoO_*x*_/CoSn_2_ after OER (top right) and crystalline CoSn_2_ after HER (bottom right) with SAED inset, scale bar = 10 nm. Adapted with permission from ref. 221 copyright 2018, Wiley-VCH.

We recently investigated CoSn_2_ for overall water splitting where after alkaline OER, CoSn_2_ underwent a slow but rather complete structural transformation (Fig. 11). The loss of Sn from the structure of CoSn_2_ led to a disordered, defected, and vacant amorphous active material with a CoO_*x*_/CoOOH phase (with Co^III^ centered active species) that enables optimal adsorption of the oxygen species (O_ad_), thus facilitating the formation of adsorbed –OOH species (OOH_ad_) by a nucleophilic attack, thereby promoting the deprotonation of OOH_ad_ to produce O_2_.^221^ However, in the case of HER, only surface Sn loss was observed exposing the Co^0^ site for H^+^ adsorption (Fig. 11).

In all cases, the stability of the catalysts was also linked to the extent of structural transformations. If the transformation is very rapid and complete, no change in the stability curve is observed as the formed species are already sufficient to withhold the stable current under longer run.^105^ However, if the transformation is slower, an initial decrease in current was observed until a formation of core–shell is attained (not allowing further electrolyte permeability) and then a constant current at applied potentials is achieved.^98^ In both cases, the extent of transformation was found to be important to produce an enormous number of active sites, increased surface areas, or even electronic conductivity.

## 9. Conclusion and challenges

Intermetallic compounds have emerged as compelling advanced energy materials for HER, OER, and overall water splitting. In various examples, intermetallic catalysts are displayed to promote the challenging reactions of water splitting with enhanced activity, improved reaction kinetics, and long-lasting durability. In this perspective, we have highlighted the suitability of well-defined intermetallic (pre)catalysts for the design of high-performance electrocatalysts with complex interface structure, bonding characteristics, and electronic properties, which is essential to increase overall energetic efficiencies of the water-splitting reaction and stabilization of their active structures under operating conditions. Although substantial efforts have been devoted to exploring intermetallic compounds as novel catalysts for water splitting, they are relatively underdeveloped. Therefore, several critical aspects need to be taken into account for the further development of this field and are discussed below.

One of the essential factors for designing an efficient electrocatalyst is by synthesizing the materials in nanoscale, increasing the number of active sites, active surface areas, and electronic conductivities. The properties of intermetallic compounds are closely associated with their size, shape, structure, composition, and crystal phase and hence, they must be tuned and optimized according to the need of electrocatalysis. In order to form an intermetallic compound, a large amount of energy is required, which is usually achieved with high-temperature annealing methods. Such solid-state techniques often predictably give rise to large agglomerated particles or even by-products and in most cases, with significantly decreased catalytic active surface areas.^30^ Thus, it is extremely interesting to design low-temperature novel synthetic strategies that can allow better atomic-level control over the stoichiometry, high dispersity of nanostructures with access to unique electronic and surface structures. In this regard, low-cost techniques such as wet impregnation, SSP, chemical reduction, and electrochemical approaches entail more attention. Similarly, looking at the various known classes of intermetallic compounds with different element combinations, a knowledge-guided simulation could also be highly important for their rational synthesis with desired HER and OER properties.^28^

Most importantly, it is now well-known that most of the intermetallic catalysts transform (either step-wise or completely) from their pristine state to active state under catalytic operating HER and OER conditions. Therefore, understanding the dynamic behavior of the catalyst through advanced *ex situ* and *operando* techniques to reveal the real active species, morphological and electronic changes, surface/bulk structure, and structure–activity relation during catalysis is of utmost significance. Indeed, efforts should also be devoted to testing the active intermetallic catalysts for OER and HER for a longer period at higher currents to observe their chemical stability. This will not only will help us to understand the reaction mechanism but also to further optimize the catalyst design with desired elements.

As the current studies have demonstrated the applicability of intermetallic compounds for electrocatalytic water splitting, it is now essential to examine them (either unifunctional or bifunctional) in industrially relevant conditions at elevated temperatures as well as at higher current densities in harsh alkaline or acidic environments. This step is pivotal, as the reaction conditions set for the lab-scale are entirely different when compared to the actual working electrolyzers. In addition to this, emphasis should also be given to the stability and the degradation of catalysts under operating conditions. Thus, future studies should be focussed on designing nanostructured intermetallic electrocatalysts with specific structure-types with porosity, precise control in their particle size and morphology with higher surface areas, enhanced electronic conductivity, structures with electrolyte permeability and bulk-activity, fast-redox switching sites, the maximum amount of edge sites, which have already been proven beneficial for non-oxidic materials.^16,237^ Like-wise, such designed intermetallics are expected to work under high current densities, elevated temperature, all-pH electrolytes, and for seawater splitting. In the near future, the research should also be concentrated on producing large electrodes with excellent mechanical and (electro)chemical stability, resistance to acidic/alkaline media, and bifunctionality.

The tunability of intermetallic materials by varying different metals could make this class of materials also interesting for electrocatalytic applications beyond water-splitting. In this context, we recently combined the electrochemical water-splitting and selective (almost 100%) oxygenation of organic substrates through *in situ* surface modification of intermetallic FeSn_2_ precatalyst. Selective oxygenation is a demanding approach and bears higher economic value than oxygen produced by water splitting. Therefore, intermetallic catalysts could be used to further explore this nascent field where H_2_ and oxygenated products can be liberated simultaneously, making it a lucrative technology. Alternatively, the same electrochemical technology with intermetallics can be applied in non-aqueous solvents to drive the other essential transformations such as regeneration of the triphenylphosphine from triphenylphosphine oxide as well as a one-pot Wittig olefination reaction that presently best driven by noble-metal catalysts.^238^

Furthermore, the scope of the intermetallics can easily be extended electrochemical carbon dioxide (CO_2_) reduction, a promising reaction to mitigate CO_2_ emissions, where presently transition metals (in particularly Cu) have been used predominantly to yield high-end multicarbon products. Notably, the intermetallics can also be of special interest for the kinetically sluggish oxygen reduction reaction (ORR), an important cathodic reaction of fuel cell which is presently mediated by the metals, alloys, and carbons, *etc.* Another alternative strategy would be to utilize intermetallics to achieve a systematic electrochemical reduction of heavy non-metal oxides with very strong E–O Bonds (E = Si, P, S).^239^ Besides, intermetallics have also shown promising electrocatalytic behavior methanol oxidation reaction,^240^ surface coatings,^241^ and supercapacitors. Although intermetallics is relatively an unexplored field for electrocatalysis, more fundamental and applied research pursued simultaneously to uncover novel classes of materials and study their intriguing unusual properties toward energy applications.

## Author contributions

C. W. conducted the literature research and wrote the first draft of the manuscript. P. W. M. initiated the overall idea and contributed to the planning and writing of the manuscript. M. D. and P.W. M. discussed, supervised, and edited the final version of the perspective.

## Conflicts of interest

There are no conflicts to declare.
